# Machine Learning Force Field for Optimization of Isolated and Supported Transition Metal Particles

**DOI:** 10.1021/acs.jctc.4c01606

**Published:** 2025-02-25

**Authors:** Alexandre Boucher, Cameron Beevers, Bertrand Gauthier, Alberto Roldan

**Affiliations:** † Cardiff Catalysis Institute, School of Chemistry, University of Cardiff, Main Building, Park Pl, Cardiff CF10 3AT, U.K.; ‡ School of Mathematics, Cardiff University, Abacws Building, Senghennydd Rd, Cardiff CF24 4AG, U.K.

## Abstract

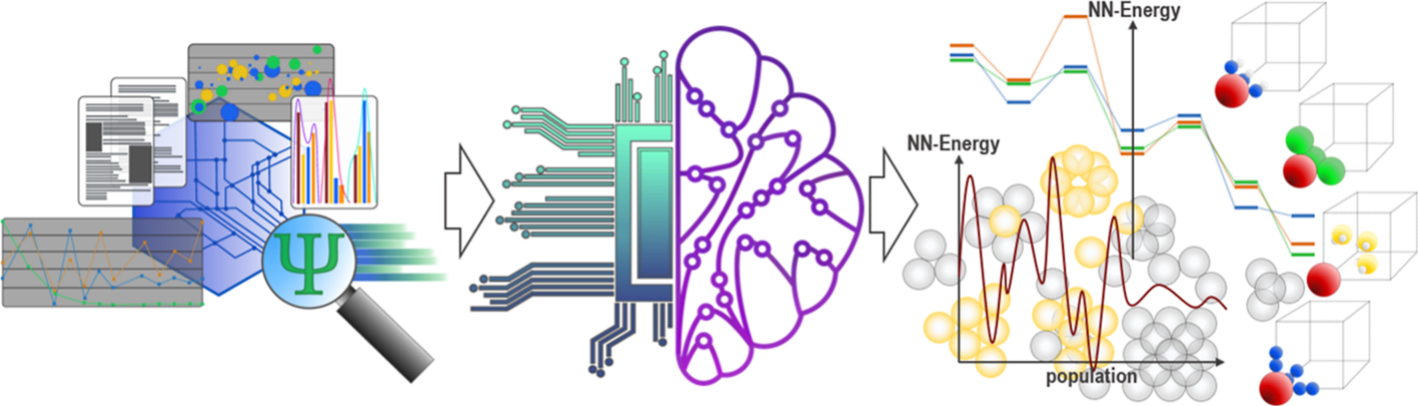

Computational modeling is an integral part of catalysis research. With it, new methodologies are being developed and implemented to improve the accuracy of simulations while reducing the computational cost. In particular, specific machine-learning techniques have been applied to build interatomic potential from ab initio results. Here, we report an energy-free machine-learning calculator that combines three individually trained neural networks to predict the energy and atomic forces of metallic particles. The investigated structures were a monometallic Pd nanoparticle, a bimetallic AuPd nanoalloy, and supported Pd metal crystallites on silica. Atomic energies were predicted via a graph neural network, leading to a mean absolute error (MAE) within 0.004 eV from density functional theory (DFT) calculations. The task of predicting atomic forces was split over two feed-forward networks, one predicting the force norm and another its direction. The force prediction resulted in a MAE within 0.080 eV/Å against DFT results. The interpretability of the graph neural network predictions was demonstrated by underlying the physics of the monometallic particle in the form of cohesion energy.

## 1 Introduction

Many phenomena, such as the arrangement of metal atoms in gas-phase or supported metal particles, their interactions with a surface, or with substrates in a reactive environment, are governed by complex atomic interactions. The development of metal-based materials is instrumental to many industry sectors, e.g. energy^1−3^ and environmental control.^4^ Since its development in the 1960s,^5^ the density functional theory (DFT) has become the workhorse of condensed matter theoretical research and an instrumental tool to enhance and guide the development of metal-based technologies.

Developing these materials, e.g. metal-based catalysts or high-performance alloys,^6−13^ requires exploring chemical systems that are too complex to be treated by DFT. Instead, classical force fields, e.g. Sutton-Chen, Finnis-Sinclair, or Gupta potentials,^14−16^ have been developed with the aim of reducing the computational resources necessary to explore these chemical spaces. Although widely employed over the past decades,^17−21^ these force fields’ accuracy does not reach that of *ab initio* calculations, e.g. DFT.^22−24^ Furthermore, the parametrization and formulation of these force fields, based on the bulk properties of the material, make them fundamentally unable to describe accurately the energies and forces of small particles or systems involving an interface between the metal and another material as is the case of metal nanocoating or supported metal particles with a large ration of undercoordinated atoms.^25−27^

The recent progress in predicting potential energy surfaces (PES) based on representative data sets of spanned chemical space interpreted through machine learning (ML) architectures led to neural network interatomic potentials (NNIPs) with near-DFT accuracy.^28−35^ ML has proven to be a powerful tool for accelerating computational research.^28,29,34,36−41^ NNIPs allow for highly efficient computation at a cost up to 1000 times cheaper than an accurate DFT calculation.

The present work introduces an innovative tool for predicting the energies and forces of individual atoms forming isolated and supported metal clusters. The method introduced reaches the so-called near-accuracy at a computational cost several orders of magnitude lower than that of DFT and provides a reliable tool for the exploration of the chemical space associated with a collection of particles with different shapes and sizes, known as a metastable ensemble.^26,41,43^

Our approach combines state-of-the-art graph neural networks (GNN) in an energy-free approach, where distinct neural networks compute the atomic energies independently from the forces. The investigated systems are gas-phase palladium and AuPd bimetallic clusters and supported palladium clusters on α-silica. The predictions reached near-DFT accuracy at a small fraction of its computational cost. The resulting set of neural networks predicting atomic energies and forces was organized in a specific architecture called a machine-learning calculator (ML-calculator). The ML-calculator forms an autonomous tool that can be coupled with existing DFT algorithms to accelerate the calculation rate or work on its own as an independent NNIP for geometry optimization.^25,26,43−69^

## 2 Method

### 2.1 Density Functional Theory Calculations

All calculations performed to generate the required data sets, i.e., geometry optimizations of Pd-pure and AuPd-alloy clusters in gas-phase and Pd supported on hydroxylated α-SiO_2_(001), a widely employed support material in the catalyst production industry,^42^ were carried out using spin-polarized density-functional theory (DFT) as implemented in the Vienna Ab initio Simulation Package (VASP).^70−72^ The revised PBE functional from Perdew, Burke, and Ernzerhof (RPBE) was used to calculate the exchange–correlation energy.^73,74^ The projected-augmented wave (PAW) pseudopotentials were employed to describe core electrons.^72,75^ Dispersion corrections were included through Grimme’s dispersion correction scheme, DFT-D3.^76^ The plane-wave kinetic cutoff was set to 500 eV, the electronic energy convergence threshold set to 1 × 10^–7^ eV, and the ionic convergence to 0.04 eV/Å. Gaussian smearing was employed to describe the distribution of electrons around the Fermi level, with a smearing parameter of 0.2 eV for pure metallic structures and 0.1 eV for SiO_2_ surfaces and supported clusters.

Supported Pd NPs on the α-SiO_2_(001) surface were modeled using a *p*(2 × 2) supercell preventing the interactions of supported metal atoms with periodic images. The silica support contained 3 SiO_2_ layers, and all surface dangling bonds were saturated with hydroxyl groups. Only the surface hydroxyl groups and the Pd cluster were relaxed during geometry optimizations. A vacuum layer of at least 10.0 Å was placed perpendicularly to the surface. Calculations were performed using a *k*-points density of 0.2 points/Å. Details on the setup and its justification are provided in Supporting Information, Section S1.

### 2.2 Data Sets

Creating a reference data set representative of the targeted PES is crucial to training a machine-learning neural network (NN). Due to the cost of running DFT calculations, the data set size should be kept as small as possible while ensuring the integrity of the chemical environments relevant to the PES. Three data sets were prepared in the present work: Pd-pure, AuPd-alloy, and Pd-silica data sets. Neural networks are known to extrapolate poorly beyond the points included in the data set used to train them,^81^ and therefore, it is paramount to include structures outside the potential energy minima to improve the NN’s versatility and capability, particularly for tasks such as geometry optimization: each metal structure included in the data set were either shrunk by a factor 0.8 or stretched by a factor 1.2 to ensure that, upon geometry relaxation through DFT, the PES around the equilibrium position is explored and represented in each network trained in this work.

#### 2.2.1 Pd-Pure

The data set covers gas-phase isolated Pd structures containing up to 55 atoms. It contains a total of 439 distinct structures taken from the literature and built from chemical intuition.^59,77−80^

#### 2.2.2 AuPd-Alloy

The data set contains gas-phase structures with sizes ranging from 17 to 34 atoms of 116 Pd-pure and 45 Au-pure clusters derived from the literature.^82,83^ Besides, the data set includes 93 structures of bimetallic AuPd gas-phase with various ratios with 19–27 atoms in random, Janus, and core–shell arrangements.

#### 2.2.3 Pd/SiO_2_(001)

The data set combines the gas-phase Pd-pure data set (439 structures) with 66 structures of up to 8 Pd atoms supported on the α-silica slab. It also contains 7 silica structures derived from the pristine α-silica (001) surface: a fully hydroxylated surface, a shrunk along the axis perpendicular to the slab, another two with only top –OH groups shrunk and elongated, and the last ones with one, two and three –OH groups missing.

### 2.3 Capturing the Atomic Environment

The atomic environment of each atom in the data set was converted into a processable vector via a procedure known as fingerprinting. Over the years, several flavors have been developed based on, for instance, atom-centered symmetry functions or smooth overlap of atomic position (SOAP).^84−86^ To predict the properties at local (atomic energies and forces) and global (total energy) levels, the fingerprint must satisfy the following characteristics:

i) Translational and rotational invariance, i.e., the predicted property does not change upon translation or rotation of the entire system in space;
ii) Uniqueness, i.e., it captures each distinct atomic environment in a fashion that prevents degenerated representation of different atoms;
iii) Computational efficiency, i.e., the time to compute it must remain minimal against the time needed to calculate reference data points;
iv) Completeness, i.e., it captures the most relevant features. The completeness of the different fingerprints employed by the community is an ongoing debate and an active field of research.^87^

The fingerprint developed in the present work includes local and nonlocal data from the atomic structure. Local information was obtained using the *G*^2^ and *G*^3^ symmetry functions introduced by J. Behler, respectively capturing radial and angular features.^88,89^ These functions have been extensively described in the literature and successfully employed to generate multiple neural network interatomic potentials (NNIPs).^29,31−33,86,88,89^ Nonlocal information was expressed through the *G*^2^ and *G*^3^ functions combined with the Chebyshev polynomials basis, respectively relabeled  and  as described in Table 1. Nonlocal functions are referred to in this work as perturbed symmetry functions. These functions capture atomic distances and angles centered on all neighbors of the target atom and provide the network with additional information regarding 2-bodies and 3-bodies features existing around the target atom, i.e., they depend on a cutoff radius.^90^ The cutoff function employed in this work is the cosine function introduced by Behler and given in eq 1(88)
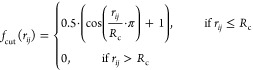
1

**Table 1:** Construction of the Perturbed Symmetry Functions Used to Fingerprint Non-Local Information^a^

nonlocal radial function	nonlocal angular function
*G*^2^ symmetry-function	*G*^3^ symmetry-function
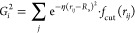	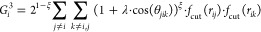
hyperparameters: η, *R*_s_	hyperparameters: ξ, λ
first-order Chebyshev polynomials	second-order Chebyshev polynomials
*T*_0_(*x*) = 1	*U*_0_(*x*) = 1
*T*_1_(*x*) = *x*	*U*_1_(*x*) = 2*x*
***T***_***l***+1_(***x***) = 2*x*·*T*_*l*_(*x*) – *T*_*l–*1_(*x*)	*U*_*l*_(*x*) = 2*x*·*T*_*l*_(*x*) – *U*_*l–*1_(*x*)
	from second-order polynomials, we build first-order pseudopolynomials, *T*_*l*+1/2_
	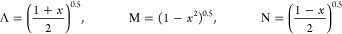
	***T***_***l***+1/2_(***x***) = Λ·*T*_*l*_(*x*) – *ΜΝ*·*U*_*l–*1_(*x*)
perturbed *G*^2^ symmetry-function	perturbed *G*^3^ symmetry-function
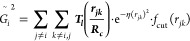	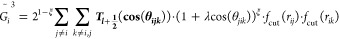
hyperparameters: η, *l*, *R*_c_ nonlocal information	hyperparameters: ξ, λ, *l*, *R*_c_ nonlocal information

a The hyperparameter η controls the width of the Gaussian function described by the *G*^2^ function, and *R*_s_ centres the Gaussian at the specified distance away from the atom target of the fingerprint. In the *G*^3^ function, λ determines whether the cosine function is centered on 0 or π, and ξ controls the width of the angular function. In the non-local functions, *R*_c_ is the cut-off radius of the fingerprint, and *l* determines the order of the Chebyshev polynomial employed.

The prediction of atomic forces was decomposed into two parts: amplitude and direction. The force amplitude is translation and rotation invariant; therefore, the symmetry and perturbed symmetry functions can be used for their predictions. The directional fingerprint, **G**^**D**^, was used to determine the direction of the force. It is based on the *G*^2^ symmetry function and described in eq 2(35)
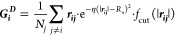
2where |***r***_***ij***_| represents the Euclidian norm of the vector ***r***_***ij***_ from the fingerprinted atom *i* to its neighbor *j*. The number *N*_*j*_ represents the number of neighbors accounted for, and the factor 1/*N*_*j*_ is used to normalize the fingerprint.

To complete the fingerprint, information relevant to the chemical nature of the environment around each atom was captured through a collision-free weighting approach introduced by Beevers et al.^91^ According to number theory, any given natural integer, , can be expressed as a product of prime factors raised by an appropriate exponent in a unique fashion, as shown in eq 3.
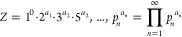
3where *p*_*n*_ are successive prime numbers and *a*_*n*_ are the appropriate exponents. Passing eq 3 to the logarithmic, prime numbers form the basis of a vector space where the exponents are its coefficients, as described in eq 4.
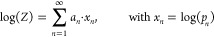
4

**Figure 1:**
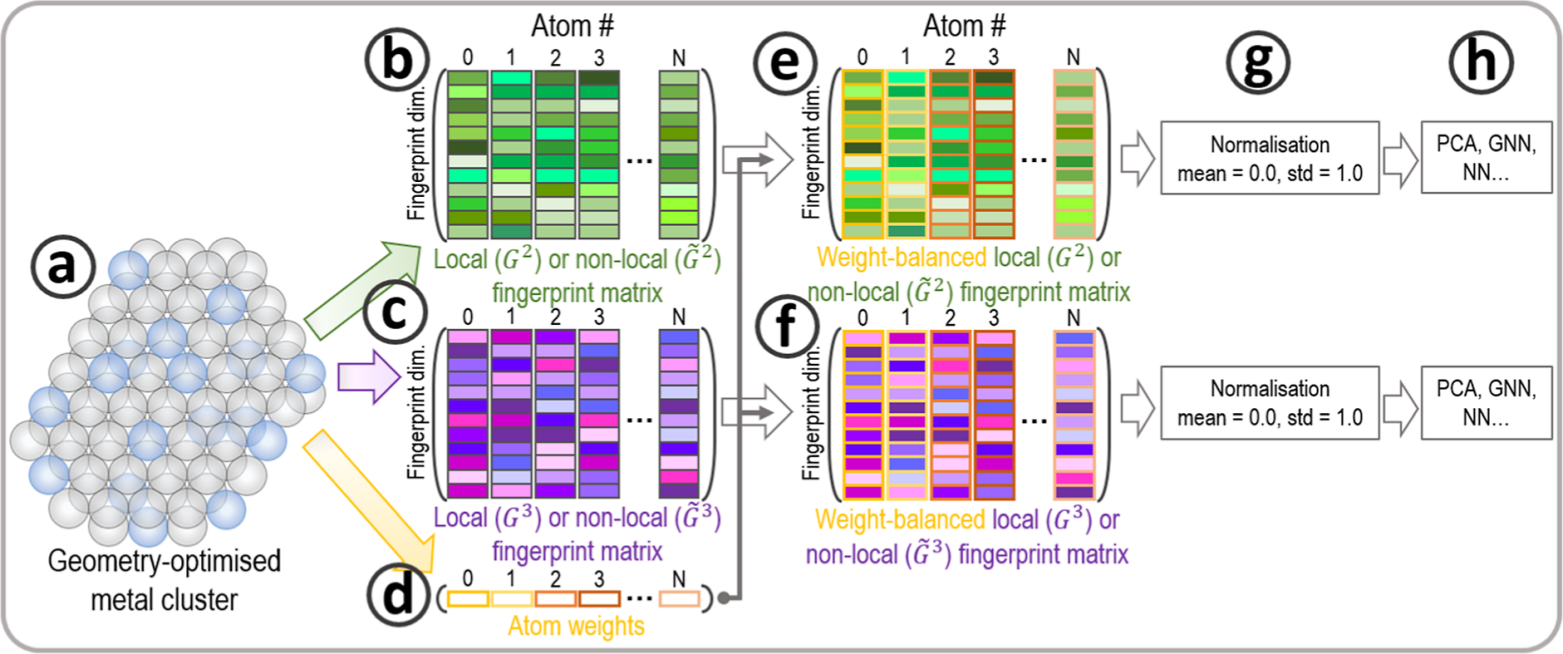
Workflow of extracting the fingerprint of each atom in the system and applying atomic weight. From (a) DFT-optimized particles, the symmetry functions are employed to extract fingerprint matrices (b,c) and weight vectors for each atom (d). Each atomic weight is then applied to the fingerprint matrices (e,f), and those matrices are normalized (g) and fed to ML algorithms (h).

The chemical nature of elements in the fingerprint was described by associating them with a distinctive prime number, forming a basis set of the form ***X*** = {*p*_1_, *p*_2_, ..., *p*_*K*_} for *K*-elements in the structure. Thus, the fingerprint reflected both the atom’s nature and the nature of its closest neighbors. The weight associated with each fingerprint was calculated through eq 5.
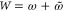
5where ω is the on-site weight, ω = *p*_*i*_, where *p*_*i*_ is the prime number associated with the atom’s element in the basis set ***X***, and ω̃ is the neighbor’s weight contribution calculated using eq 4 as the sum of the logarithm of the prime number associated with the element of each neighbor within the cutoff radius around the targeted atom. The fingerprinting procedure is sketched in Figure 1.

Three different sets of fingerprints were used in this work. Table 2 shows the symmetry functions *G*^2^ and *G*^3^ applied on each data set; Table 3 shows the parameters used for the  and  perturbed functions. Finally, the same fingerprint was applied to each data set for the direction-covariant fingerprint, given in Table 4. The initial parameters were chosen similar to parameters in the literature and iteratively corrected to include the additional terms until the best accuracy is reached.^28,32,33,92^

**Table 2:** Detailed of the *G*^2^ and *G*^3^ Symmetry Functions Employed on Each Dataset Understudied in This Work

function	weights	*R*_s_ [Å]	η [Å2]	λ [Ø]	ξ [Ø]
Pd-Pure & AuPd-Alloy Data Sets					
*G*^2^	none (Pd-pure); Pd: 2, Au: 3 (AuPd-nanoalloy)	[2.5, 3.0, 3.5, 4.0, 4.5, 5.0, 5.5, 6.0]	[0.5, 1.0, 3.0, 6.0]	n.a.	n.a.
*G*^3^	none (Pd-pure); Pd: 2, Au: 3 (AuPd-nanoalloy)	n.a.	n.a.	[+1, −1]	[1.0, 2.0, 4.0, 8.0, 16.0]
Pd-Silica Data Set					
*G*^2^	H: 2, O: 3, Si: 5, Pd: 7	[3.0, 3.5, 4.0, 4.5, 5.0, 5.5, 6.0]	[1.0, 3.0, 6.0]	n.a.	n.a.
*G*^3^	H: 2, O: 3, Si: 5, Pd: 7	n.a.	n.a.	[+1, −1]	[2.0, 8.0, 16.0]

**Table 3:** Detailed of the  and  Symmetry Functions Employed on Each of the Datasets Understudied in This Work

function	weights	*R*_s_ [Å]	η [Å^2^]	Cheby. order	λ [Ø]	ξ [Ø]	Pseudo-Cheby. order
Pd-Pure & AuPd-Alloy Data Sets							
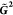	none (Pd-pure); Pd: 2, Au: 3 (AuPd-nanoalloy)	0.0	[1.0, 3.0, 6.0]	[2, 4, 6]	n.a.	n.a.	n.a.
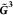	none (Pd-pure); Pd: 2, Au: 3 (AuPd-nanoalloy)	n.a.	n.a.	n.a.	[+1, −1]	[1.0, 4.0, 8.0, 16.0]	[3, 4, 5]
Pd-Silica Data Set							
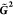	H: 2, O: 3, Si: 5, Pd: 7	0.0	[1.0, 3.0, 6.0]	[2, 4, 6]	n.a.	n.a.	n.a.
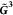	H: 2, O: 3, Si: 5, Pd: 7	n.a.	n.a.	n.a.	[+1, −1]	[1.0, 8.0, 16.0]	[3, 4, 5]

**Table 4:** Detailed of the **G**^**D**^ Function Employed on all of the Datasets Understudied in This Work

	weight	*R*_s_ (Å)	η (Å^2^)	λ (Ø)	ξ (Ø)
*G*^D^	none	[2.5, 3.0, 3.5, 4.0, 4.5, 5.0, 5.5, 6.0]	[1.0, 3.0, 6.0]	Ø	Ø

### 2.4 Building the Neural Networks

#### 2.4.1 Graphical Neural Network: Energy Prediction

The data used in this work covered a wide variety of cluster shapes and sizes, leading to different fingerprint arrays, i.e., nontabular data, which GNN can handle conveniently. The developed GNN required three elements to predict atomic energies: The fingerprint matrix, **M**_**e**_, which describes the atom’s environments; the adjacency matrix, **A**, which represents the node connections within the graph; and the atom-weight matrix, **W**, which captures the element of each atom. The reader is referred to the PyTorch and PyTorch-Geometric libraries documentation for details on the different parameters used to build the networks.^93,94^Table 5 reports the different GNN structures employed on each data set studied in this work.

**Table 5:** Detailed Structure of the GNN Built for Energy Predictions

GNN structure	optimizer	weight and biases
Pd-pure: 150–150–150–150–150–1^a^	Pd-pure: NAdam, L2-regularization strength: 0.5 × 10^–3^, 660 epochs.	Pd-pure, AuPd-alloy: weights: Xavier uniform, bias:
AuPd-alloy: 400–150–150–150–150–1	AuPd-alloy: NAdam, L2-regularization strength: 0.5 × 10^–3^, 960 epochs	
Pd/silica: 150–150–150–150–150–150–1	Pd/silica: NAdam, L2-regularization strength: 10^–3^, 1200 epochs	Pd/Silica: weights: Kaiming normal, bias:
no message passing. global-add-pool readout function activation functions: RReLU (Pd-pure and Pd/silica), Leaky-ReLU (AuPd-alloy).		

a Indicates the number of neurons in each layer, i.e. a first, second, third, fourth, and fifth layer containing 150 neurons and an output layer of 1 neuron.

**Figure 2:**
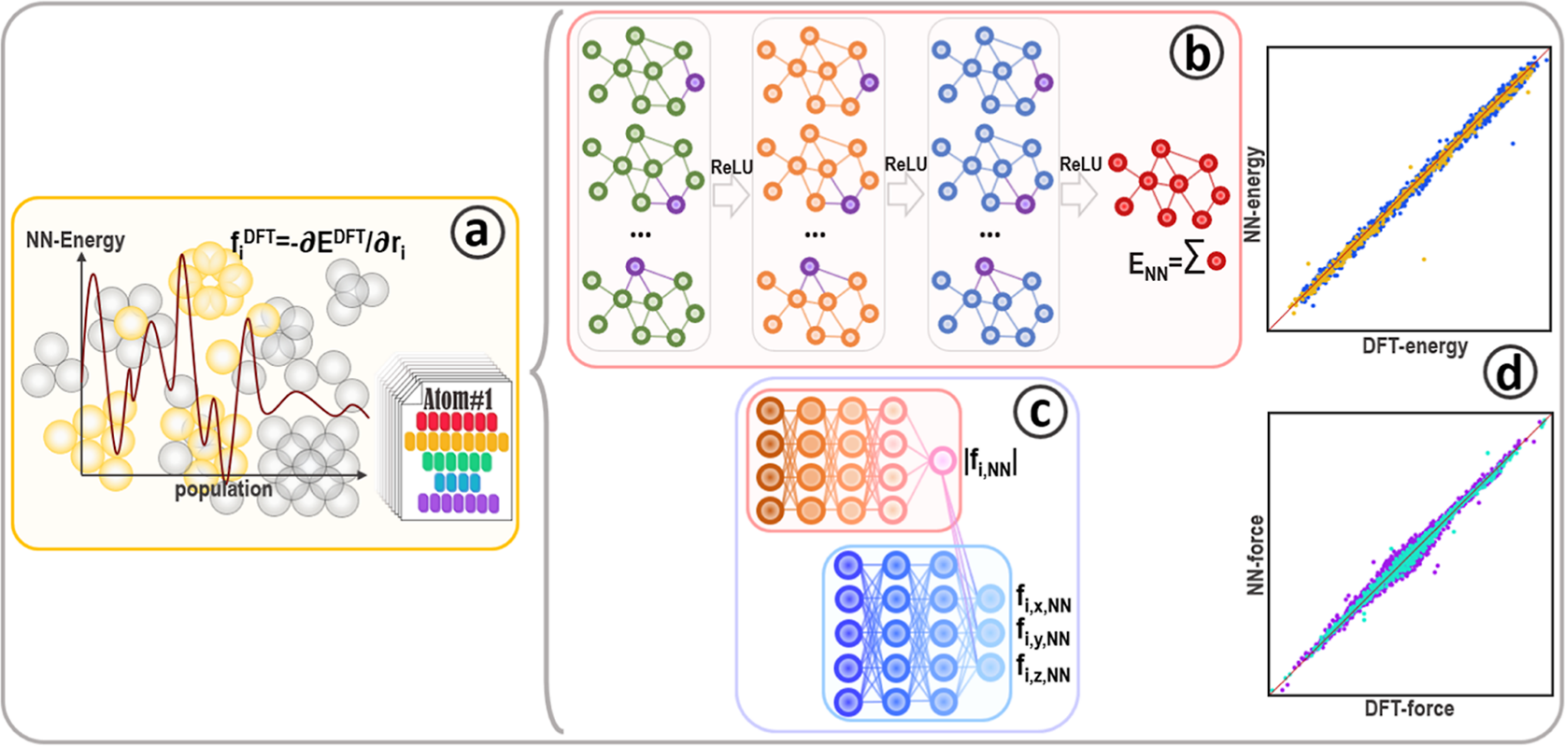
Schematic workflow of the predictive ML-calculator introduced in this work. (a) The data set is generated. The data set stores DFT total energies and atomic forces as targets, and the fingerprint of each atom is extracted. (b) Each fingerprint is assembled into a graph. Each node in the graph represents an atom, and a connection between two nodes represents a chemical bond. The GNN predicts atomic and total energies. (c) The second set of NNs predicts the Euclidian norm of the force acting on each atom and its direction. (d) The predictions are compared against the data set data.

The predicted total energy of a cluster was calculated as a sum of individual atomic contributions independently of the cluster’s shape, size, nature, or state (gas-phase or supported). All data sets were split into 80% for training and 20% for validation during the learning process. From the training set, 16% of the total data set was used for on-the-fly validation, avoiding overfitting. The NNIPs defined in the present work were built according to the workflow depicted in Figure 2 and described as follows:

a) the chemical space is sampled using DFT, data sets are created, and the fingerprints are constructed;
b) a graph neural network (GNN) is employed to predict atomic energies. Each node in the graph corresponds to a cluster atom with an associated energy. A readout function sums the energy predicted on each node according to eq 6, where ε_*n*_ is the value attached to one node (atom) in the graph:

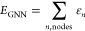
6

#### 2.4.2 Feed-Forward Neural Network: Forces Prediction

Classic feed-forward networks were built to predict the forces’ norms and directions, as illustrated in Figure 2. The NNs’ structures are described in Tables 6 and 7.

**Table 6:** Detailed Structure of the Feed-Forward NNs Built for Forces-Norm Predictions^a^

feed-forward NN structure	optimizer	weight and biases
Pd-pure and AuPd-alloy: 280–80–60–1. Activation function: RReLU	all data sets: NAdam, L2-regularization strength: 0.5 × 10^–3^, 1200 epochs.	all data sets: weights: Xavier normal, bias:
Pd/silica: 80–40–40–1. Activation function: Leaky-ReLU		

a The softplus activation function was applied to the output layers of each NN.

**Table 7:** Detailed Structure of the GNN Built for Energy Predictions

feed-forward NN structure	optimizer	weight and biases
all data sets: 400–120–100–100–100–3, activation functions: Leaky-ReLU (all data sets).	all data sets: NAdam, L2-regularization strength: 10^–4^, 1000 epochs.	all data sets: weights: Xavier uniform, bias: zeros

## 3 Results

### 3.1 Data Preprocessing

#### 3.1.1 Near-Equilibrium Structures

The quality of the data employed during training is intrinsically linked to the quality of the prediction, and therefore, great care was taken regarding the data set’s preprocessing. In particular, the DFT cluster optimization led to a significant proportion of the data set describing quasi-identical near-equilibrium structures. In order to avoid overweighting quasi-identical images in the data sets, a filter that operates on each trajectory step was designed. The filter recursively compares the energy per atom of consecutive images. If the energy difference falls under a threshold, the image is ignored and not included in the data set (details given in Supporting Information, Section S2).

For the gas-phase data sets, an energy threshold of 3 × 10^–3^ eV/atom was chosen to capture the diversity of the spanned structures in the fingerprint. On supported Pd/SiO_2_ clusters, due to the complexity of this chemical space and rigidity of the support, a lower threshold of 1 × 10^–3^ eV/atom was set to include a higher number of atomic environments close to the equilibrium. For consistency, the Pd-pure gas-phase clusters included in the Pd–SiO_2_(001) data set also used the same threshold.

#### 3.1.2 Fingerprint Noise Reduction

In the scope of this work, the fingerprinting procedure can be considered as an information channel converting information from the atomic simulation environment (ASE) object into a tensor object. This procedure may introduce noise in the data that can perturb the learning procedure and hinder the performance of the ML-based algorithm.^95^ To reduce the influence of the noise on the data’s quality, different preprocessing methods were tested to improve the NN’s predictions. Two commonly employed methods were compared, principal component analysis (PCA) and autoencoders (AE), which proved powerful preprocessing tools. Their details and results are described in the Supporting Information, Section S3.^96,97^ In brief, PCA builds linear relationships between components by projecting a matrix of dimension [*N* × *D*] to a new matrix [*N* × *D*^′^], where *N* represents the number of atoms in a cluster, *D* the initial dimension of the fingerprint, and each dimension *D*′ represents a linear component derived from the initial *D*-dimensional fingerprint with *D*′ < *D*. AE follows the same principle as PCA but through a structure similar to neural networks, building more complex relationships than linear components as PCA does. Whereas PCA remains deterministic, AE must be trained to reduce the initial fingerprint’s dimension efficiently, leading to the loss of noise in the original data. The most significant influence of preprocessing was observed in predicting the norm of forces. The results obtained using PCA and AE were compared only on Pd/SiO_2_ using the fingerprints described in Tables 2 and Table 3.

**Figure 3:**
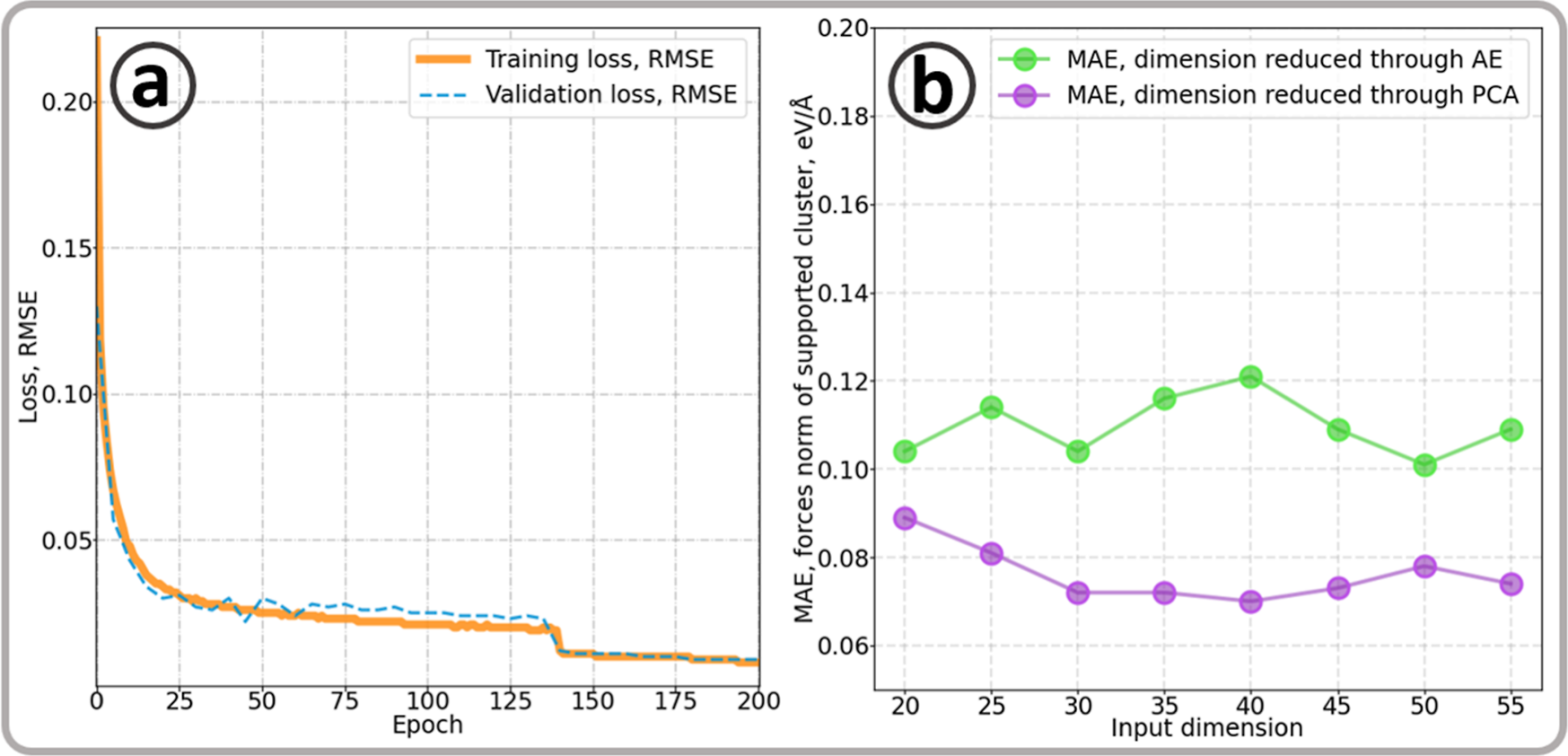
(a) The learning curve of the AE to reduce the dimension of the forces norm fingerprint from 60D to 30D. (b) The mean average error (MAE) of the neural network predicting the atomic forces norm with different preprocessing through autoencoding (AE) and principal component analysis (PCA). RMSE and MAE are in eV/atom.

The results obtained after the reduction of the initial 60D fingerprint indicate better performance from PCA by 0.02–0.03 eV/Å on average against AE, as illustrated in Figure 3. An improvement of 0.05 eV/Å against the baseline result obtained without preprocessing the initial 60D fingerprint. The AE preprocessing performs the encoding-decoding procedure with minimal error, as shown by the AE learning curve in Figure 3a, where a RMSE below 0.01 eV/atom was reached after just 200 epochs, indicating that the encoding-decoding procedure is performed with minimal loss of information through data-compression, i.e. the dimension-reduced vector contains all the relevant information stored in the initial vector. However, despite this, PCA produced better results and was employed in this work to preprocess the fingerprint associated with forces norm.

### 3.2 ML-Calculator: Energy Predictions

**Figure 4:**
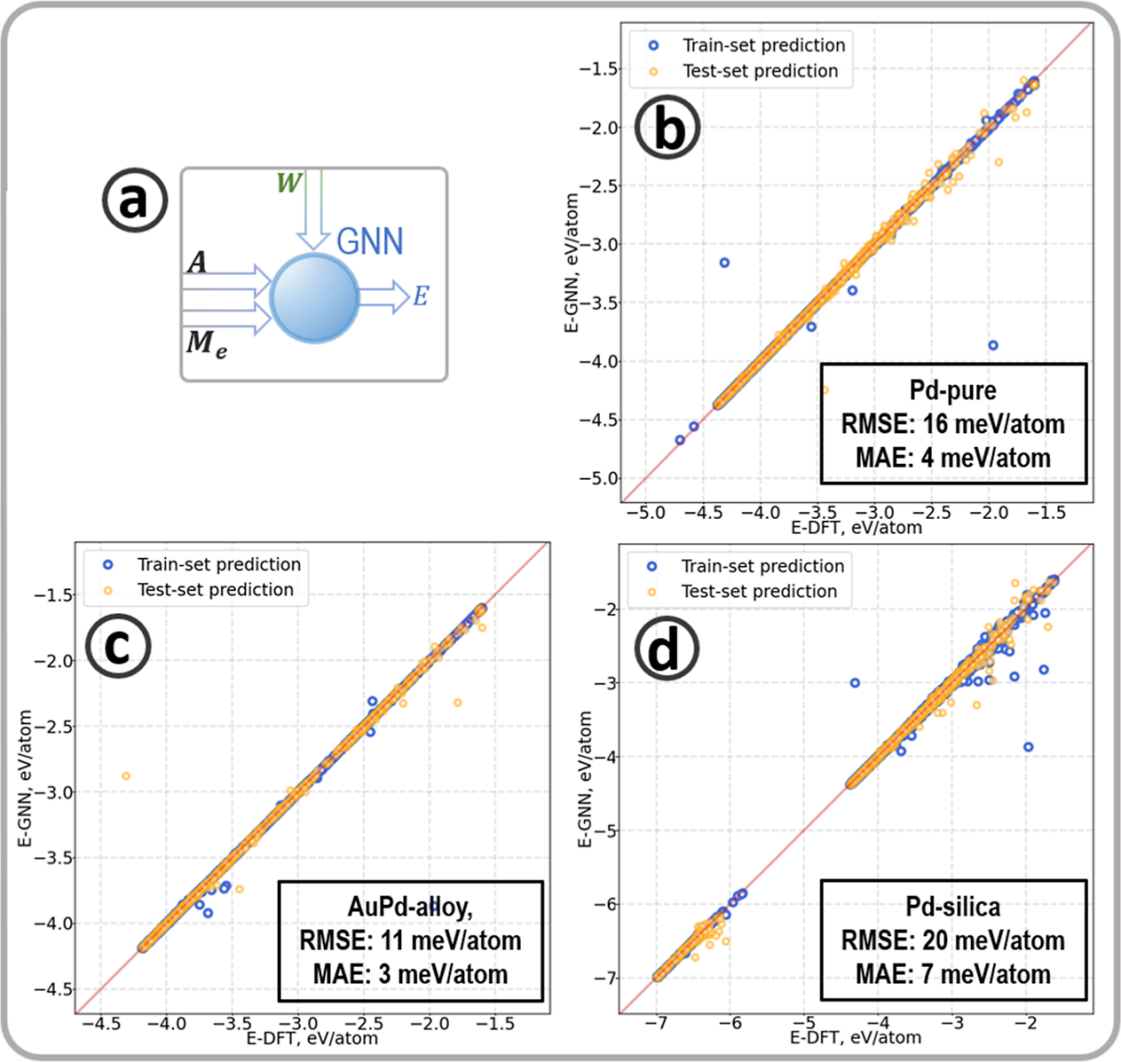
(a) Illustration of the part of the ML-calculator predicting the energy. The GNN requires the adjacency matrix (**A**), the energy fingerprint (**M**_**e**_), and atom weights (**W**). (b–d) show the correlation between predicted and DFT-energies for Pd-pure, AuPd-alloy, and Pd-silica data sets.

Figure 4 shows the energy predictions and the mean absolute errors (MAE) for the three systems: Gas-phase Pd and AuPd nanoparticles and Pd/α-SiO_2_(001). The MAE ranges between 0.003 and 0.007 eV/atom and follows the order AuPd-alloy (gas) < Pd-pure (gas) < Pd/SiO_2_, as shown in Table 8. The most significant errors in the predicted energies correspond to unstable structures whose geometries are far from any minima in the potential energy surface.

**Table 8:** Predictions Accuracy in Forces Norm and Total Vectorial Forces for the Three Datasets Understudied in This Work^a^

data set	δ***E***, eV/atom	number of entries	energy error, MAE, eV/atom	energy error, RMSE, eV/atom
gas-phase Pd-pure	0.003	15,500	0.004	0.016
gas-phase AuPd-alloy	0.003	11,000	0.003	0.011
gas-phase and supported Pd/SiO_2_	0.001	25,000	0.007	0.020

a δ*E* is the energy threshold used in the pre-processing filter.

**Figure 5:**
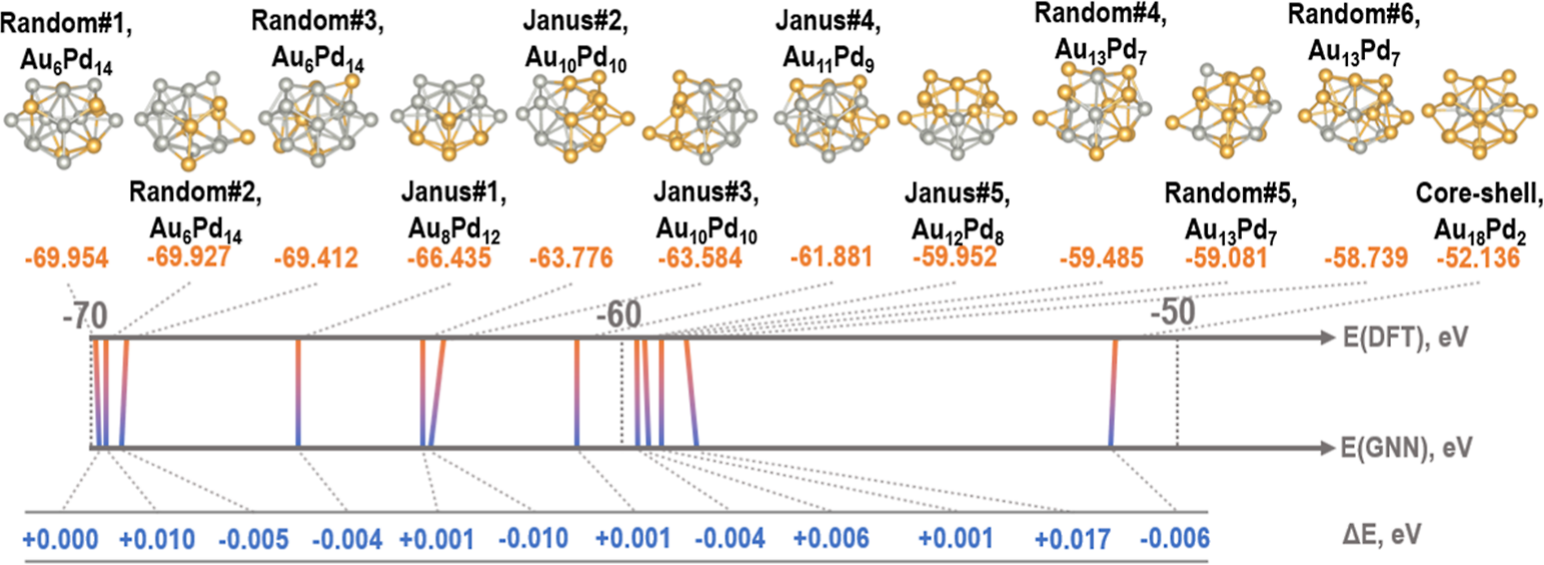
Diagrammatic representation of the bimetallic clusters containing 20 atoms. The DFT and GNN predicted total energies and their difference, Δ*E*(GNN), are in eV over the entire tested cluster, i.e. eV/cluster.

It is worth mentioning the flexibility of the ML-calculator in accurately predicting the energy of alloy particles from a relatively small data set containing actual multimetallic structures. Figure 5 compares the predicted and DFT-calculated total energies of 12 particles containing 20 atoms with compositions from Au_6_Pd_14_ to Au_18_Pd_2_ with Random, Janus, and core–shell configurations. The GNN predicts total errors from 0.001 to 0.017 eV/cluster.

### 3.3 ML-Calculator: Forces Predictions

**Figure 6:**
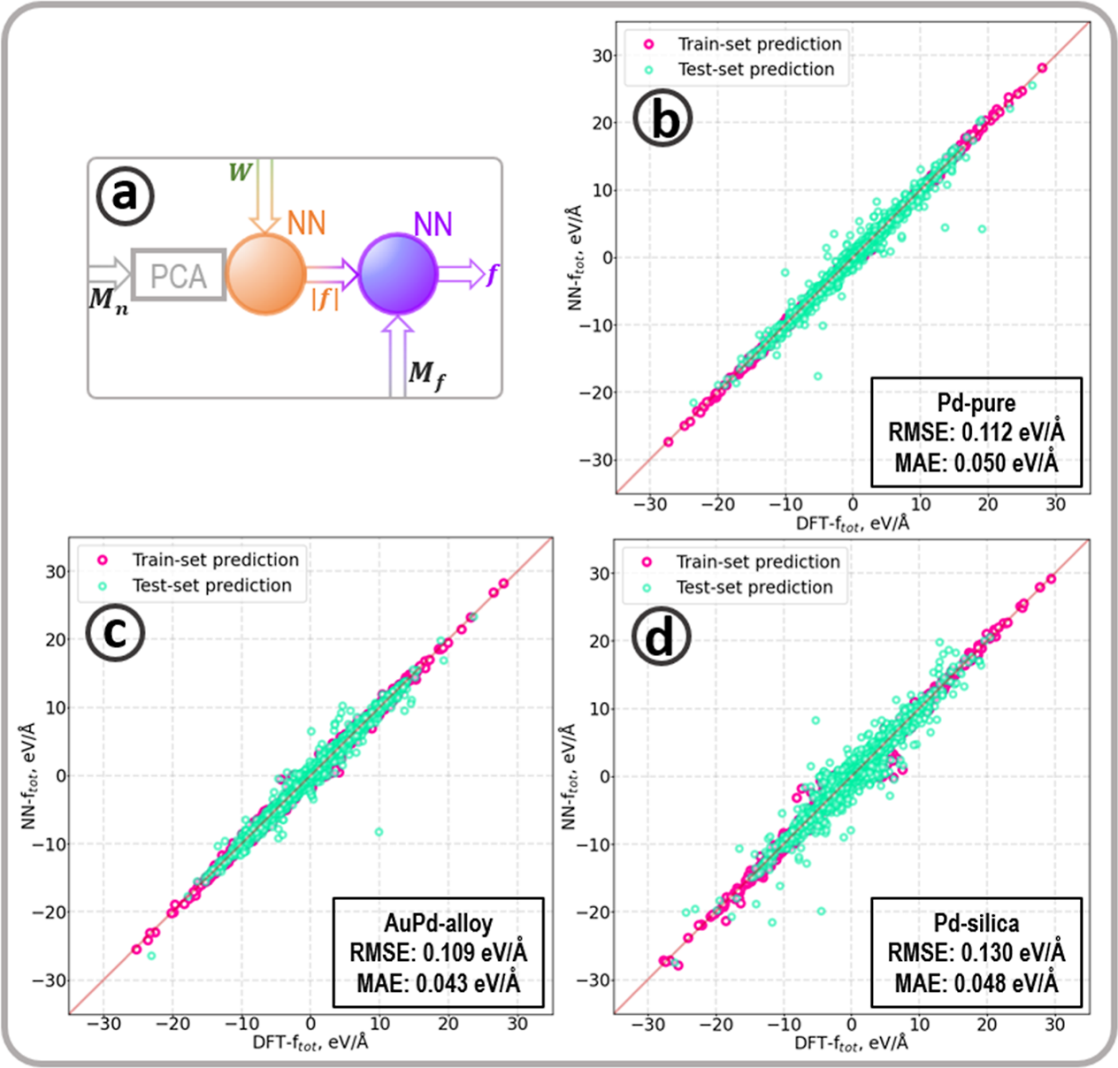
(a) Illustration of the part of the ML-calculator predicting atomic forces. A first feed-forward NN predicts the forces’ norm using the atomic weights multiplied by the PCA-reduced fingerprint, **M**_**n**_. A second feed-forward NN predicts the total force using the direction fingerprint for input, **M**_**f**_, and multiplying the 3D output with the predicted forces norm. (b–d) show the correlations between DFT and predicted forces for the Pd-pure, AuPd-alloy, and Pd-silica-data set.

Atomic force is the product of the force norm and the direction vector. Figure 6a describes the PCA-transformed forces-norm generated from a first NN feeding a second feed-forward NN for predicting the vectorial forces. The accuracies of the predictions are summarized in Table 9, where δ*E* represents the energy threshold to select two images from the same optimization path (near-equilibrium preprocessing), MAE is the mean absolute error, and RMSE is the root mean squared error. Predictions of the vectorial forces consistently show an improvement compared to the forces norm, as illustrated in Table 9 by reducing the MAE by 37%, 22%, and 44% for the Pd-pure, AuPd-alloy, and Pd/silica data sets, respectively. This observation can be explained mathematically: The norm is multiplied with a 3D vector whose components are inferior or equal to 1 (direction unit vector), generating smaller values. Furthermore, because the direction vector is trained independently from the norm, the second NN can undirectly learn a correction to the force norm in each of the 3 vectorial coordinates and apply it to the norm before the loss is calculated.

**Table 9:** Predictions Accuracy in Forces Norm and Total Vectorial Forces for the Three Datasets Understudied in This Work^a^

data set	δ***E***, eV/atom	number of entries	norm MAE, eV/Å	vectorial force MAE, eV/Å	vectorial force RMSE, eV/Å
gas-phase Pd-pure	0.003	240,000	0.080	0.050	0.112
gas-phase AuPd-alloy	0.003	150,000	0.055	0.043	0.109
gas-phase and supported Pd/SiO_2_	0.001	500,000	0.086	0.048	0.120

a δ*E* is the energy threshold used in the pre-processing filter.

In the chosen structure of two embedded feed-forward NNs, the rotation-covariant direction NN acts as the unit-vector prediction and corrects the forces-norm. It is also noticeable in Table 9 that the more accurate the “guess” on forces norm, the higher the accuracy on the resultant vectorial force. Figure 6b–d shows the graphical comparison between predicted and DFT forces for the three systems understudy. The accuracy reached on all of the data sets studied in this work lies within the so-called near-DFT accuracy, referring to a level of accuracy where the trained force field can compete with DFT, usually considered to be reached in the literature for a force RMSE around or under 0.100 eV/Å.^22,28,32,33,92,98^

### 3.4 Interpretability of the Results

The GNN is trained to provide a set of atomic energies with the only restriction that the sum of energies should approach the DFT energy of the system under study. This raises the question of the interpretability of each atomic contribution to the total energy; does the GNN learn the “physics” associated with the data set, or is the predicted atomic energy a mathematical artifact with no physical value?

**Figure 7:**
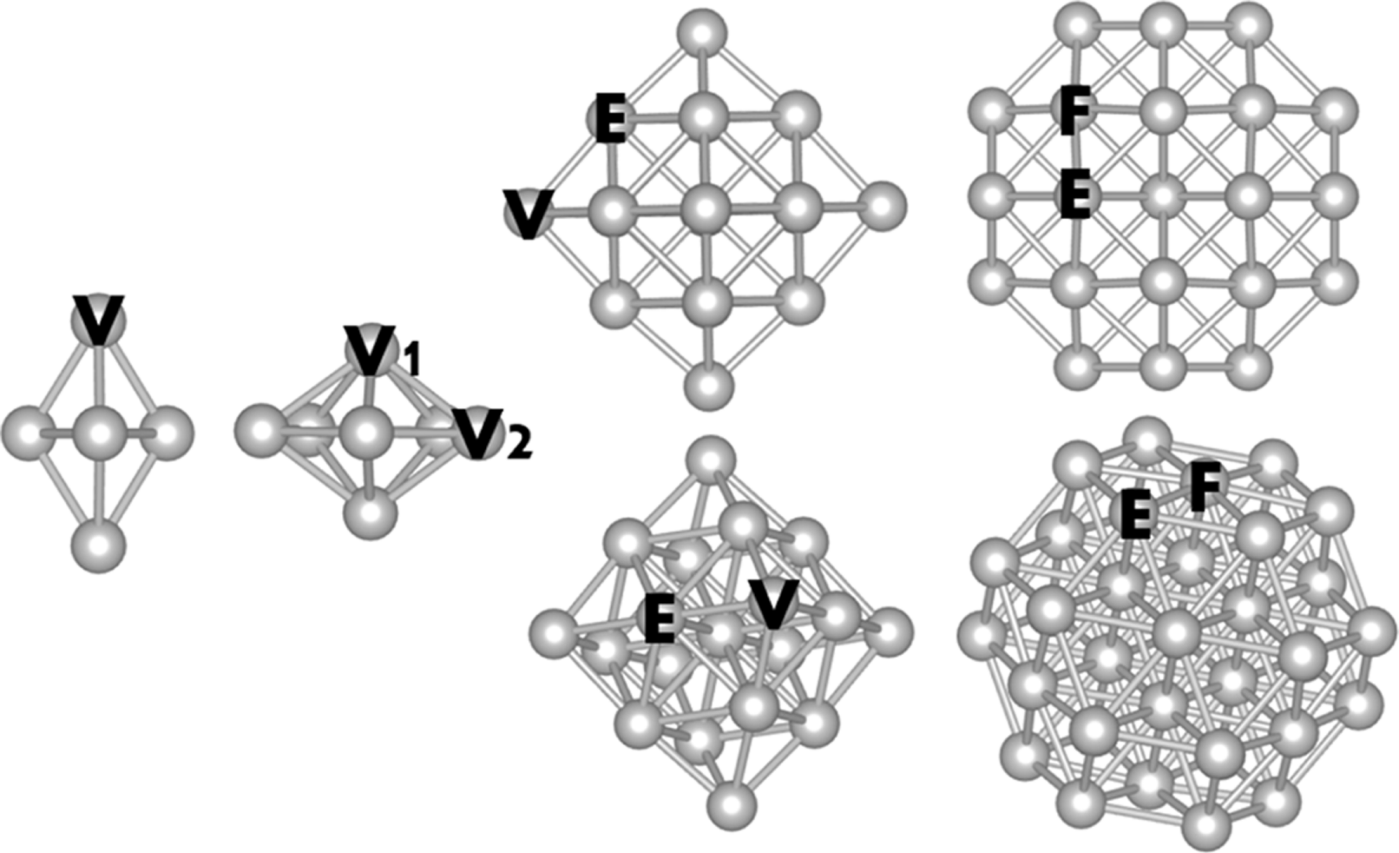
Representation of the four Pd-pure clusters selected to evaluate the interpretability of the energy predictions. From left to right: Pd_5_, Pd_7_, Pd_19_, and Pd_38_. The initials V, E, and F indicate the position of the atoms selected to investigate their cohesion energy on the vertex, edge, and facet.

Four Pd-pure gas-phase clusters, illustrated in Figure 7, were chosen to predict the cohesion energy of specific atoms in the vertex, edges, and facets. The energy variation of pulling individual atoms away from the metal cluster can be approached with a Morse potential curve,^99,100^ the general equation is in eq 8.
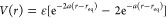
8where *r*_e_ represents the equilibrium distance between the target atom and the cluster, ε is the potential reached at the bottom of the Morse curve well and quantifies the strength of the interaction between the atom and the rest of the cluster, i.e., the cohesion energy, *E*_coh_. The parameter *a* expresses the width of the well and is related to the stiffness of the interaction, *k*_e_, at the bottom of the well, . In addition, the norm of the force vector predicted by the NN should equal the derivative of the Morse curve, expressed in eq 9.
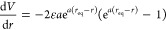
9

**Figure 8:**
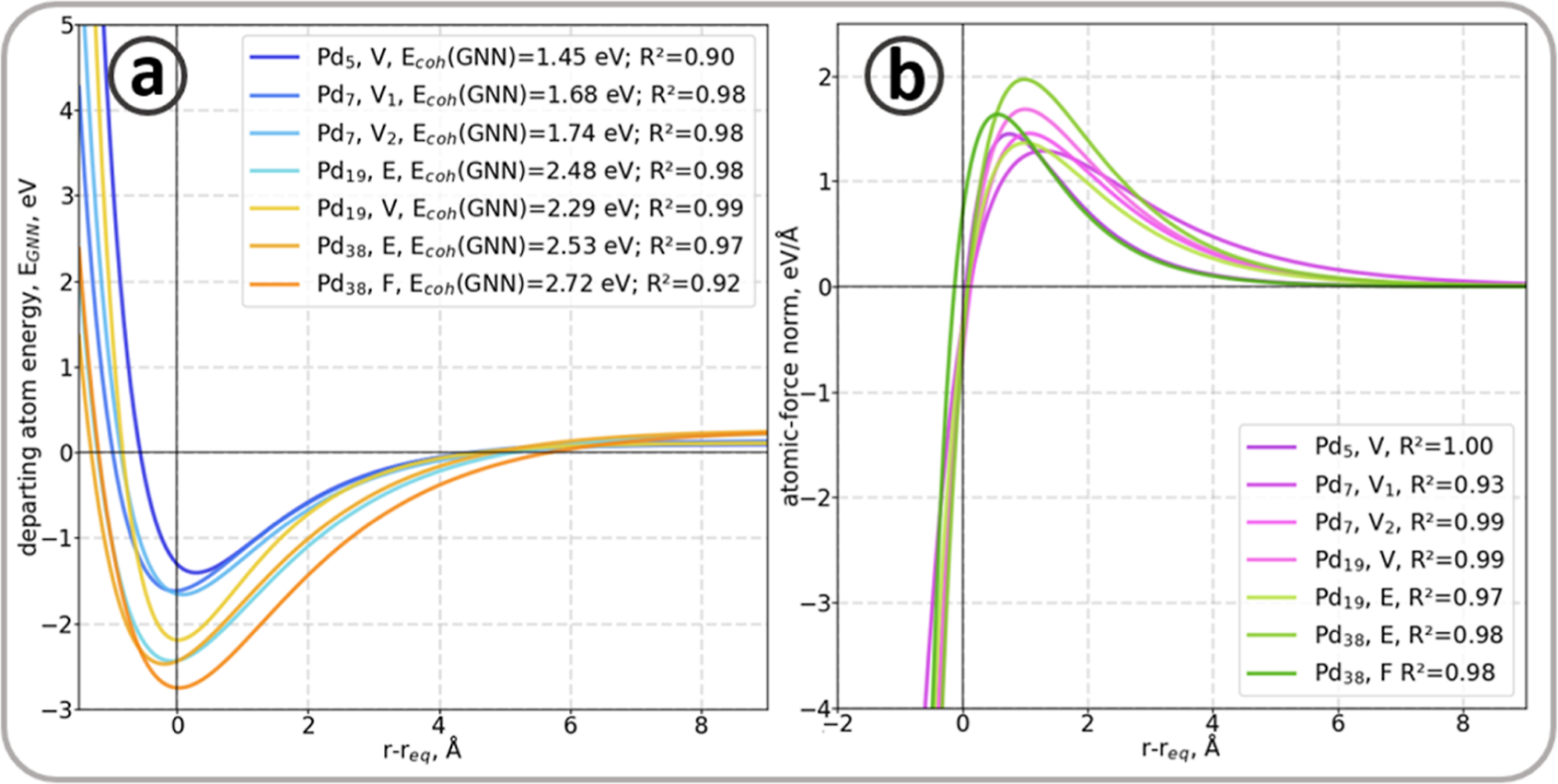
(a) Cohesion energy and (b) force’s norm of the targeted atoms in Pd_*x*_ clusters (*x* = 5, 7, 19, 38) as a function of the atom-cluster distance.

The distances between the targeted atoms and the clusters were systematically increased, and the energies and forces were predicted. Figure 8a represents the predicted energies following the Morse curves, i.e. with coefficients of determination (*R*^2^) at least 0.9. The meaning of correlation between predicted energies and the Morse potential indicates that the GNN learned the underlying physics carried by the atomic fingerprints. In other words, an interpretable physical meaning can be associated with each contribution to the total energy predicted by the GNN. The cohesion energies predicted by the GNN are in good agreement with values reported in the literature, with errors under 0.02 eV, demonstrating the applicability of the algorithm introduced in the present work.^101,102^ They also agree well with the cohesion energy calculated from DFT, i.e. 1.52, 1.71, 2.30, and 2.59 eV for Pd_5_, Pd_7_, Pd_19_, and Pd_38_, respectively.

The evaluation of the predicted forces norm also shows good agreement (*R*^2^ ≥ 0.9) with the expected trend given by eq 9 and represented in Figure 8b, demonstrating the interpretability of the ML-calculator.

## 4 Conclusion

The work presents an improved atomic cluster fingerprinting that is able to capture local and nonlocal and the nature of atoms matter, easing the use of advanced computational techniques in physical science, particularly nanoscience and catalysis. The fingerprint feeds three machine learning structures to accurately predict atomic and cluster energies, atomic forces norm and direction. Following an energy-free approach, these grap- and feed-forward networks were combined in an autonomous machine-learning calculator.^35^ This calculator was tested against gas-phase Pd, AuPd, and Pd/SiO_2_ clusters, representing contemporary challenges to designing multimetallic and supported catalysts. Analysis of the energy and forces predictions revealed a near DFT accuracy for the different systems. Besides, the atomic energy interpretability was tested and confirmed to encapsulate physical meaning, such as the cohesion energy. Overall, the innovative ML-calculator is accurate and highly flexible, producing competitive results or better than existing neural networks’ interatomic potentials (NNIPs).^28,32,33,103−108^

## Supporting Information Available

The Supporting Information adjunct to this paper contains the (i) setup for DFT parameters applied to construct the different data sets used in the present work, (ii) a detailed description of the data filters applied to the raw data, i.e. VASP output files, and (iii) a section outlining the preprocessing and accuracy of data reduction methods such as the principal component analysis and the autoencoders (PDF)

## References

[ref1] Cui C. H.; Yu S. H. Engineering interface and surface of noble metal nanoparticle nanotubes toward enhanced catalytic activity for fuel cell applications. Acc. Chem. Res. 2013, 46, 1427–1437. 10.1021/ar300254b. 23425040

[ref2] Yin Z.; Lin L.; Ma D. Construction of Pd-based nanocatalysts for fuel cells: Opportunities and challenges. Catal. Sci. Technol. 2014, 4, 4116–4128. 10.1039/C4CY00760C.

[ref3] Antolini E. Palladium in fuel cell catalysis. Energy Environ. Sci. 2009, 2, 915–931. 10.1039/b820837a.

[ref4] Wang W. H.; Himeda Y.; Muckerman J. T.; Manbeck G. F.; Fujita E. CO2 Hydrogenation to Formate and Methanol as an Alternative to Photo- and Electrochemical CO2 Reduction. Chem. Rev. 2015, 115, 12936–12973. 10.1021/acs.chemrev.5b00197. 26335851

[ref5] Hohenberg P.; Kohn W. Inhomogeneous electron gas. Phys. Rev. 1964, 136, B864–B871. 10.1103/PhysRev.136.B864.

[ref6] George E. P.; Raabe D.; Ritchie R. O. High-entropy alloys. Nat. Rev. Mater. 2019, 4, 515–534. 10.1038/s41578-019-0121-4.

[ref7] Ye Y. F.; Wang Q.; Lu J.; Liu C. T.; Yang Y. High-entropy alloy: challenges and prospects. Mater. Today 2016, 19, 349–362. 10.1016/j.mattod.2015.11.026.

[ref8] Zhang Y.; et al. Microstructures and properties of high-entropy alloys. Prog. Mater. Sci. 2014, 61, 1–93. 10.1016/j.pmatsci.2013.10.001.

[ref9] Singh R.; Sharma A.; Singh P.; Balasubramanian G.; Johnson D. D. Accelerating computational modeling and design of high-entropy alloys. Nat. Comput. Sci. 2021, 1, 54–61. 10.1038/s43588-020-00006-7. 38217165

[ref10] Liu J.; Bunes B. R.; Zang L.; Wang C. Supported single-atom catalysts: synthesis, characterization, properties, and applications. Environ. Chem. Lett. 2018, 16, 477–505. 10.1007/s10311-017-0679-2.

[ref11] James T. E.; Hemmingson S. L.; Campbell C. T. Energy of Supported Metal Catalysts: From Single Atoms to Large Metal Nanoparticles. ACS Catal. 2015, 5, 5673–5678. 10.1021/acscatal.5b01372.

[ref12] Maniam K. K.; Chetty R.; Thimmappa R.; Paul S. Progress in the Development of Electrodeposited Catalysts for Direct Liquid Fuel Cell Applications. Appl. Sci. 2022, 12, 501 10.3390/app12010501.

[ref13] Okamoto K.; Akiyama R.; Yoshida H.; Yoshida T.; Kobayashi S. Formation of nanoarchitectures including subnanometer palladium clusters and their use as highly active catalysts. J. Am. Chem. Soc. 2005, 127, 2125–2135. 10.1021/ja047095f. 15713089

[ref14] Sutton A. P.; Chen J. Long-range Finnis–Sinclair potentials. Philos. Mag. Lett. 1990, 61, 139–146. 10.1080/09500839008206493.

[ref15] Ackland G.; Sutton A.; Vitek V. Twenty five years of Finnis–Sinclair potentials. Philos. Mag. 2009, 89, 3111–3116. 10.1080/14786430903271005.

[ref16] Gupta R. P. Lattice relaxation at a metal surface. Phys. Rev. B:Condens. Matter Mater. Phys. 1981, 23, 6265–6270. 10.1103/physrevb.23.6265.

[ref17] Gomez L.; Dobry A.; Diep H. Melting properties of fcc metals using a tight-binding potential. Phys. Rev. B:Condens. Matter Mater. Phys. 1997, 55, 6265 10.1103/PhysRevB.55.6265.

[ref18] Kallinteris G.; Papanicolaou N.; Evangelakis G.; Papaconstantopoulos D. Tight-binding interatomic potentials based on total-energy calculation: Application to noble metals using molecular-dynamics simulation. Phys. Rev. B:Condens. Matter Mater. Phys. 1997, 55, 2150–2156. 10.1103/physrevb.55.2150.

[ref19] Willaime F.; Massobrio C. Temperature-induced hcp-bcc phase transformation in zirconium: A lattice and molecular-dynamics study based on an N-body potential. Phys. Rev. B:Condens. Matter Mater. Phys. 1989, 63, 2244–2247. 10.1103/physrevlett.63.2244. 10040837

[ref20] Massobrio C.; Pontikis V.; Martin G. Molecular-dynamics study of amorphization by introduction of chemical disorder in crystalline NiZr2. Phys. Rev. B:Condens. Matter Mater. Phys. 1990, 41, 10486–10497. 10.1103/physrevb.41.10486. 9993456

[ref21] Lai W.; Liu B. Influence of interfacial texture and asymmetric growth in diffusion-limited amorphization in Ni-Zr multilayers upon medium-temperature annealing. Phys. Rev. B:Condens. Matter Mater. Phys. 1998, 58, 6063–6073. 10.1103/physrevb.58.6063.

[ref22] Zhou C.; et al. Force field for copper clusters and nanoparticles. J. Comput. Chem. 2009, 30, 2255–2266. 10.1002/jcc.21210. 19263432

[ref23] Zhang Q.; Tang E.; Xi Y.; Han B.; Legenski N.; Chalas G.; Chan F.; Cheng H.; Forrey R. C. Analytic Force Field for Clusters and Nanoparticles of Aluminum and Its Hydride. Phys. Rev. Appl. 2014, 1, 054004 10.1103/physrevapplied.1.054004.

[ref24] Zhang X. Q.; et al. Site stability on cobalt nanoparticles: A molecular dynamics reaxff reactive force field study. J. Phys. Chem. C 2014, 118, 6882–6886. 10.1021/jp500053u.

[ref25] Carchini G.; et al. How theoretical simulations can address the structure and activity of nanoparticles. Top. Catal. 2013, 56, 1262–1272. 10.1007/s11244-013-0093-3.

[ref26] Zandkarimi B.; Alexandrova A. N. Dynamics of Subnanometer Pt Clusters Can Break the Scaling Relationships in Catalysis. J. Phys. Chem. Lett. 2019, 10, 460–467. 10.1021/acs.jpclett.8b03680. 30633531

[ref27] Abdeen D. H.; El Hachach M.; Koc M.; Atieh M. A. A Review on the Corrosion Behaviour of Nanocoatings on Metallic Substrates. materials 2019, 12, 210 10.3390/ma12020210. 30634551 PMC6356964

[ref28] Paleico M. L.; Behler J. Global optimization of copper clusters at the ZnO(1010) surface using a DFT-based neural network potential and genetic algorithms. J. Chem. Phys. 2020, 153. 10.1063/5.0014876. 32770878

[ref29] Behler J.; Parrinello M. Generalized neural-network representation of high-dimensional potential-energy surfaces. Phys. Rev. Lett. 2007, 98, 146401 10.1103/physrevlett.98.146401. 17501293

[ref30] Nitol M. S.; Dickel D. E.; Barrett C. D. Artificial neural network potential for pure zinc. Comput. Mater. Sci. 2021, 188, 110207 10.1016/j.commatsci.2020.110207.

[ref31] Behler J. Four Generations of High-Dimensional Neural Network Potentials. Chem. Rev. 2021, 121, 10037–10072. 10.1021/acs.chemrev.0c00868. 33779150

[ref32] Weinreich J.; Römer A.; Paleico M. L.; Behler J. Properties of α-Brass Nanoparticles. 1. Neural Network Potential Energy Surface. J. Phys. Chem. C 2020, 124, 12682–12695. 10.1021/acs.jpcc.0c00559.

[ref33] Liu M.; Kitchin J. R. SingleNN: Modified Behler-Parrinello Neural Network with Shared Weights for Atomistic Simulations with Transferability. J. Phys. Chem. C 2020, 124, 17811–17818. 10.1021/acs.jpcc.0c04225.

[ref34] Ouyang R.; Xie Y.; Jiang D. E. Global minimization of gold clusters by combining neural network potentials and the basin-hopping method. Nanoscale 2015, 7, 14817–14821. 10.1039/C5NR03903G. 26308236

[ref35] Mailoa J. P.; et al. A fast neural network approach for direct covariant forces prediction in complex multi-element extended systems. Nat. Mach. Intell. 2019, 1, 471–479. 10.1038/s42256-019-0098-0.

[ref36] Schreiner M.; Bhowmik A.; Vegge T.; Jørgensen P. B.; Winther O. NeuralNEB—neural networks can find reaction paths fast. Mach Learn. Sci. Technol. 2022, 3, 045022 10.1088/2632-2153/aca23e.

[ref37] Ye W.; Chen C.; Wang Z.; Chu I. H.; Ong S. P. Deep neural networks for accurate predictions of crystal stability. Nat. Commun. 2018, 9, 3800 10.1038/s41467-018-06322-x. 30228262 PMC6143552

[ref38] Huang Y.; Kang J.; Goddard W. A.; Wang L. W. Density functional theory based neural network force fields from energy decompositions. Phys. Rev. B 2019, 99, 064103 10.1103/physrevb.99.064103.

[ref39] Li H.; Shi L.; Zhang M.; Su Z.; Wang X.; Hu L.; Chen G. Improving the accuracy of density-functional theory calculation: The genetic algorithm and neural network approach. J. Chem. Phys. 2007, 126, 144101 10.1063/1.2715579. 17444695

[ref40] Yang P. J.; Sugiyama M.; Tsuda K.; Yanai T. Artificial Neural Networks Applied as Molecular Wave Function Solvers. J. Chem. Theory Comput. 2020, 16, 3513–3529. 10.1021/acs.jctc.9b01132. 32320233

[ref41] Zhai H.; Alexandrova A. N. Ensemble-Average Representation of Pt Clusters in Conditions of Catalysis Accessed through GPU Accelerated Deep Neural Network Fitting Global Optimization. J. Chem. Theory Comput. 2016, 12, 6213–6226. 10.1021/acs.jctc.6b00994. 27951667

[ref42] Soled S. Silica-supported catalysts get a new breath of life. Science 2015, 350, 1171–1172. 10.1126/science.aad2204. 26785461

[ref43] Zandkarimi B.; Alexandrova A. N. Surface-supported cluster catalysis: Ensembles of metastable states run the show. Wiley Interdiscip. Rev.: Comput. Mol. Sci. 2019, 9, e1420 10.1002/wcms.1420.

[ref44] Lu Y.; Chen W. Sub-nanometre sized metal clusters: From synthetic challenges to the unique property discoveries. Chem. Soc. Rev. 2012, 41, 3594–3623. 10.1039/c2cs15325d. 22441327

[ref45] Jin R.; Higaki T. Open questions on the transition between nanoscale and bulk properties of metals. Commun. Chem. 2021, 4, 28 10.1038/s42004-021-00466-6. 36697528 PMC9814084

[ref46] Yau S. H.; Varnavski O.; Goodson T. An ultrafast look at Au nanoclusters. Acc. Chem. Res. 2013, 46, 1506–1516. 10.1021/ar300280w. 23651457

[ref47] Sohn K.; et al. Construction of evolutionary tree for morphological engineering of nanoparticles. ACS Nano 2009, 3, 2191–2198. 10.1021/nn900521u. 19621938

[ref48] Wang F.; et al. Catalytic behavior of supported Ru nanoparticles on the {100}, {110}, and {111} facet of CeO2. J. Catal. 2015, 329, 177–186. 10.1016/j.jcat.2015.05.014.

[ref49] Hutchings G. J.; Kiely C. J. Strategies for the synthesis of supported gold palladium nanoparticles with controlled morphology and composition. Acc. Chem. Res. 2013, 46, 1759–1772. 10.1021/ar300356m. 23586905

[ref50] Miyazaki A.; Balint I.; Nakano Y. Morphology Control of Platinum Nanoparticles and Their Catalytic Properties. J. Nanopart. Res. 2003, 5, 69–80. 10.1023/a:1024451600613.

[ref51] Cabié M.; et al. Direct observation of the reversible changes of the morphology of Pt nanoparticles under gas environment. J. Phys. Chem. C 2010, 114, 2160–2163. 10.1021/jp906721g.

[ref52] Simonsen S. B.; et al. Effect of particle morphology on the ripening of supported Pt nanoparticles. J. Phys. Chem. C 2012, 116, 5646–5653. 10.1021/jp2098262.

[ref53] Li D.; et al. Functional links between Pt single crystal morphology and nanoparticles with different size and shape: The oxygen reduction reaction case. Energy Environ. Sci. 2014, 7, 4061–4069. 10.1039/C4EE01564A.

[ref54] Henry C. R. Morphology of supported nanoparticles. Prog. Surf. Sci. 2005, 80, 92–116. 10.1016/j.progsurf.2005.09.004.

[ref55] Chimentão R. J.; et al. Different morphologies of silver nanoparticles as catalysts for the selective oxidation of styrene in the gas phase. Chem. Commun. 2004, 4, 846–847. 10.1039/B400762J. 15045093

[ref56] Guczi L.; et al. Modeling Gold Nanoparticles: Morphology, Electron Structure, and Catalytic Activity in CO Oxidation. J. Phys. Chem. B 2000, 104, 3183–3193. 10.1021/jp992662k.

[ref57] Khatun M.; Majumdar R. S.; Anoop A. A Global Optimizer for Nanoclusters. Front Chem. 2019, 7, 644 10.3389/fchem.2019.00644. 31612127 PMC6776882

[ref58] Hussein H. A.; Davis J. B. A.; Johnston R. L. DFT global optimization of gas-phase and MgO-supported sub-nanometre AuPd clusters. Phys. Chem. Chem. Phys. 2016, 18, 26133–26143. 10.1039/C6CP03958H. 27711424

[ref59] Davis J. B. A.; Shayeghi A.; Horswell S. L.; Johnston R. L. The Birmingham parallel genetic algorithm and its application to the direct DFT global optimization of IrN (N = 10–20) clusters. Nanoscale 2015, 7, 14032–14038. 10.1039/C5NR03774C. 26239404

[ref60] Tang Y.; Yang Z.; Dai X. A theoretical simulation on the catalytic oxidation of CO on Pt/graphene. Phys. Chem. Chem. Phys. 2012, 14, 16566–16572. 10.1039/c2cp41441d. 22806095

[ref61] Robles R.; Khanna S. N. Oxidation of Pdn (n = 1–7, 10) clusters supported on alumina/NiAl(110). Phys. Rev. B Condens Matter Mater. Phys. 2010, 82, 085428 10.1103/physrevb.82.085428.

[ref62] Cabria I.; López M. J.; Alonso J. A. Theoretical study of the transition from planar to three-dimensional structures of palladium clusters supported on graphene. Phys. Rev. B Condens Matter Mater. Phys. 2010, 81, 035403 10.1103/physrevb.81.035403.

[ref63] Sun G.; Sautet P. Metastable Structures in Cluster Catalysis from First-Principles: Structural Ensemble in Reaction Conditions and Metastability Triggered Reactivity. J. Am. Chem. Soc. 2018, 140, 2812–2820. 10.1021/jacs.7b11239. 29424224

[ref64] Zhang Z.; Zandkarimi B.; Alexandrova A. N. Ensembles of Metastable States Govern Heterogeneous Catalysis on Dynamic Interfaces. Acc. Chem. Res. 2020, 53, 447–458. 10.1021/acs.accounts.9b00531. 31977181

[ref65] Zhang Z.; Cui Z. H.; Jimenez-Izal E.; Sautet P.; Alexandrova A. N. Hydrogen Evolution on Restructured B-Rich WB: Metastable Surface States and Isolated Active Sites. ACS Catal. 2020, 10, 13867–13877. 10.1021/acscatal.0c03410.

[ref66] Baletto F. Structural properties of sub-nanometer metallic clusters. J. Phys.: Condens. Matter 2019, 31, 113001 10.1088/1361-648X/aaf989. 30562724

[ref67] Mottet C.; Goniakowski J.; Baletto F.; Ferrando R.; Treglia G. Modeling free and supported metallic nanoclusters: Structure and dynamics. Phase Transitions 2004, 77, 101–113. 10.1080/1411590310001622473.

[ref68] Engel J.; Francis S.; Roldan A. The influence of support materials on the structural and electronic properties of gold nanoparticles-a DFT study. Phys. Chem. Chem. Phys. 2019, 21, 19011–19025. 10.1039/C9CP03066B. 31465049

[ref69] Li R.; Odunlami M.; Carbonnière P. Low-lying Ptn cluster structures (n = 6–10) from global optimizations based on DFT potential energy surfaces: Sensitivity of the chemical ordering with the functional. Comput. Theor. Chem. 2017, 1107, 136–141. 10.1016/j.comptc.2017.02.010.

[ref70] Kresse G.; Hafner J. Ab initio molecular dynamics for liquid metals. Phys. Rev. B:Condens. Matter Mater. Phys. 1993, 47, 558–561. 10.1103/PhysRevB.47.558. 10004490

[ref71] Kresse G.; Furthmüller J. Efficient iterative schemes for ab initio total-energy calculations using a plane-wave basis set. Phys. Rev. B Condens Matter Mater. Phys. 1996, 54, 11169–11186. 10.1103/PhysRevB.54.11169. 9984901

[ref72] Kresse G.; Joubert D. From ultrasoft pseudopotentials to the projector augmented-wave method. Phys. Rev. B Condens Matter Mater. Phys. 1999, 59, 1758–1775. 10.1103/physrevb.59.1758.

[ref73] Perdew J. P.; Burke K.; Ernzerhof M. Generalized gradient approximation made simple. Phys. Rev. Lett. 1996, 77, 3865–3868. 10.1103/PhysRevLett.77.3865. 10062328

[ref74] Zhang Y.; Yang W. Comment on “generalized gradient approximation made simple. Phys. Rev. Lett. 1998, 80, 890 10.1103/PhysRevLett.80.890.

[ref75] Blöchl P. E. Projector augmented-wave method. Phys. Rev. B:Condens. Matter Mater. Phys. 1994, 50, 17953–17979. 10.1103/PhysRevB.50.17953. 9976227

[ref76] Grimme S.; Antony J.; Ehrlich S.; Krieg H. A consistent and accurate ab initio parametrization of density functional dispersion correction (DFT-D) for the 94 elements H-Pu. J. Chem. Phys. 2010, 132. 10.1063/1.3382344. 20423165

[ref77] Zhang W.; Xiao L.; Hirata Y.; Pawluk T.; Wang L. The simple cubic structure of Ir clusters and the element effect on cluster structures. Chem. Phys. Lett. 2004, 383, 67–71. 10.1016/j.cplett.2003.11.005.

[ref78] Waldt E.; Hehn A. S.; Ahlrichs R.; Kappes M. M.; Schooss D. Structural evolution of small ruthenium cluster anions. J. Chem. Phys. 2015, 142, 024319 10.1063/1.4905267. 25591365

[ref79] Wu X.; Sun Y. Stable structures and potential energy surface of the metallic clusters: Ni, Cu, Ag, Au, Pd, and Pt. J. Nanopart. Res. 2017, 19, 201 10.1007/s11051-017-3907-6.

[ref80] Nava P.; Sierka M.; Ahlrichs R. Density functional study of palladium clusters. Phys. Chem. Chem. Phys. 2003, 5, 3372–3381. 10.1039/B303347C.

[ref81] Dral P. O. Quantum Chemistry in the Age of Machine Learning. J. Phys. Chem. Lett. 2020, 11, 2336–2347. 10.1021/acs.jpclett.9b03664. 32125858

[ref82] Fronzi M.; Amos R. D.; Kobayashi R. Evaluation of Machine Learning Interatomic Potentials for Gold Nanoparticles—Transferability towards Bulk. Nanomaterials 2023, 13, 1832 10.3390/nano13121832. 37368262 PMC10303715

[ref83] Staykov A.; Nishimi T.; Yoshizawa K.; Ishihara T. Oxygen activation on nanometer-size gold nanoparticles. J. Phys. Chem. C 2012, 116, 15992–16000. 10.1021/jp301898t.

[ref84] Caro M. A. Optimizing many-body atomic descriptors for enhanced computational performance of machine learning based interatomic potentials. Phys. Rev. B 2019, 100, 024112 10.1103/physrevb.100.024112.

[ref85] Bartók A. P.; Kondor R.; Csányi G. On representing chemical environments. Phys. Rev. B Condens Matter Mater. Phys. 2013, 87, 184115 10.1103/physrevb.87.184115.

[ref86] Behler J.; Parrinello M. Generalized neural-network representation of high-dimensional potential-energy surfaces. Phys. Rev. Lett. 2007, 98, 146401 10.1103/physrevlett.98.146401. 17501293

[ref87] Nigam J.; Pozdnyakov S. N.; Huguenin-Dumittan K. K.; Ceriotti M. Completeness of atomic structure representations. APL Mach. Learn. 2024, 2, 016110 10.1063/5.0160740.

[ref88] Behler J. Neural network potential-energy surfaces for atomistic simulations. Chem. Model. Appl. Theory. 2010, 7, 1–41. 10.1039/9781849730884-00001.

[ref89] Behler J. Neural network potential-energy surfaces in chemistry: A tool for large-scale simulations. Phys. Chem. Chem. Phys. 2011, 13, 17930–17955. 10.1039/c1cp21668f. 21915403

[ref90] Onat B.; Ortner C.; Kermode J. R. Sensitivity and dimensionality of atomic environment representations used for machine learning interatomic potentials. J. Chem. Phys. 2020, 153. 10.1063/5.0016005. 33086812

[ref91] Beevers C.; Francis S.; Roldan A. Symmetry analysis of irregular objects. J. Math. Chem. 2023, 61, 504–519. 10.1007/s10910-022-01423-x.

[ref92] Behler J. Four generations of high-dimensional neural network potentials. Chem. Rev. 2021, 121, 10037–10072. 10.1021/acs.chemrev.0c00868. 33779150

[ref93] Paszke A. PyTorch: An Imperative Style, High-Performance Deep Learning Library. Adv. Neural Inf. Process. Syst., (2019). 32.

[ref94] Fey M., Lenssen M. Fast Graph Representation Learning with PyTorch Geometric, ArXiv Preprint 2019 arXiv:1903.02428. 10.48550/arXiv.1903.02428.

[ref95] Shannon C. E. A Mathematical Theory of Communication. Bell Syst. Tech. J. 1948, 27, 623–656. 10.1002/j.1538-7305.1948.tb00917.x.

[ref96] Arafa A.; El-Fishawy N.; Badawy M.; Radad M. RN-Autoencoder: Reduced Noise Autoencoder for classifying imbalanced cancer genomic data. J. Biol. Eng. 2023, 17, 7 10.1186/s13036-022-00319-3. 36717866 PMC9887895

[ref97] Patel H.; Upla K. P. A shallow network for hyperspectral image classification using an autoencoder with convolutional neural network. Multimed. Tool. Appl. 2022, 81, 695–714. 10.1007/s11042-021-11422-w.

[ref98] Chen C.; Deng Z.; Tran R.; Tang H.; Chu I. H.; Ong S. P. Accurate force field for molybdenum by machine learning large materials data. Phys. Rev. Mater. 2017, 1, 043603 10.1103/physrevmaterials.1.043603.

[ref99] Aldossary O. M. Generalized non-integer Lennard-Jones potential function vs. generalized Morse potential function for calculating cohesive energy and melting point of nanoparticles. J. King Saud Univ. Sci. 2021, 33, 101316 10.1016/j.jksus.2020.101316.

[ref100] Girifalco L. A.; Weizer V. G. Application of the Morse potential function to cubic metals. Phys. Rev. 1959, 114, 687–690. 10.1103/PhysRev.114.687.

[ref101] Nava P.; Sierka M.; Ahlrichs R. Density functional study of palladium clusters. Phys. Chem. Chem. Phys. 2003, 5, 3372–3381. 10.1039/B303347C.

[ref102] Nilsson J.; Carlsson P.; Grönbeck H.; Skoglundh M. First Principles Calculations of Palladium Nanoparticle XANES Spectra. Top. Catal. 2017, 60, 283–288. 10.1007/s11244-016-0612-0.

[ref103] Zhang B.; Asta M.; Wang L. W. Machine learning force field for Fe-H system and investigation on role of hydrogen on the crack propagation in α-Fe. Comput. Mater. Sci. 2022, 214, 111709 10.1016/j.commatsci.2022.111709.

[ref104] Kruglov I.; Sergeev O.; Yanilkin A.; Oganov A. R. Energy-free machine learning force field for aluminum. Sci. Rep. 2017, 7, 8512 10.1038/s41598-017-08455-3. 28819297 PMC5561031

[ref105] Li W.; Ando Y. Comparison of different machine learning models for the prediction of forces in copper and silicon dioxide. Phys. Chem. Chem. Phys. 2018, 20, 30006–30020. 10.1039/C8CP04508A. 30480270

[ref106] Chiriki S.; Bulusu S. S. Modeling of DFT quality neural network potential for sodium clusters: Application to melting of sodium clusters (Na20 to Na40). Chem. Phys. Lett. 2016, 652, 130–135. 10.1016/j.cplett.2016.04.013.

[ref107] Chiriki S.; Jindal S.; Bulusu S. S. Neural network potentials for dynamics and thermodynamics of gold nanoparticles. J. Chem. Phys. 2017, 146, 084314 10.1063/1.4977050. 28249420

[ref108] Artrith N.; Hiller B.; Behler J. Neural network potentials for metals and oxides - First applications to copper clusters at zinc oxide. Phys. Status Solidi B Basic Res. 2013, 250, 1191–1203. 10.1002/pssb.201248370.

