# Supplementary material for: Machine Learning Force Field for Optimization of Isolated and Supported Transition Metal Particles
Source: J Chem Theory Comput. 2025 Feb 25;21(5):2626–37. doi: 10.1021/acs.jctc.4c01606 (PMC11912199; doi:10.1021/acs.jctc.4c01606)
Supplement: Supplementary file 1 — ct4c01606_si_001.pdf [file ct4c01606_si_001.pdf]

A machine learning forcefield for optimization of isolated and supported transition metal particles

*Alexandre Boucher,[a] Cameron Beevers,[a] Bertrand Gauthier,[b] and Alberto Roldan[a]**

[a] *Cardiff Catalysis Institute, School of Chemistry, University of Cardiff, Main Building, Park Pl, Cardiff CF10 3AT, UK*

[b] *School of Mathematics, Cardiff University, Abacws building, Senghennydd Rd, Cardiff, CF24 4AG, UK*

corresponding author: Dr. Alberto Roldan, RoldanMartinezA@cardiff.ac.uk.

Contents

1. Calculation setups....S2
   1.1. Gas-phase particles S2
   1.2. Supported particles on silica S2
2. Data filters S4
3. Pre-processing: Principal component analysis and auto-encoding S6
4. Performance: PCA vs. AE S8
5. Reference....S10

1. Calculation setups

1.1. Gas-phase particles

Isolated gas-phase particles are described by a single k-point at the center of a vacuum box at least 10x10x10 Å large, in addition to the cluster's x, y, and z dimensions, as described in **Figure S1**. The energy is computed in the reciprocal space; therefore, a single k-point is sufficient to sample it. Dispersion corrections were accounted for through Grimme's dispersion correction scheme, DFT-D3.[73] The planewave kinetic cut-off was set to 500 eV, with an electronic energy convergence threshold set to 1×10^{-5} eV. The Gaussian smearing was employed to describe the distribution of electrons around the Fermi energy, with a smearing parameter of 0.1 eV. Particles were optimized until atomic forces felt under 0.04 eV/Å.

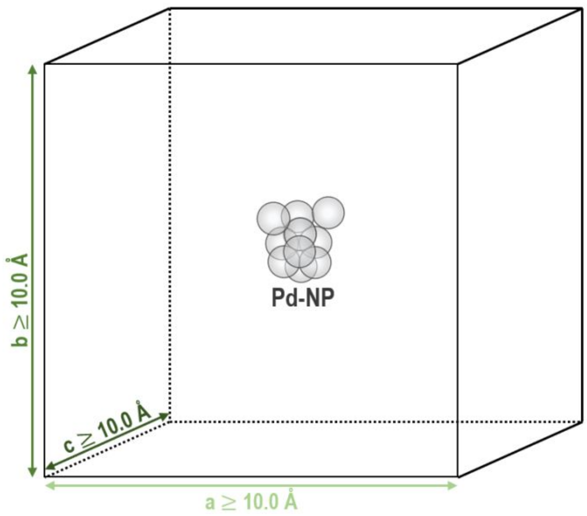

Figure S1: *Isolated palladium nanoparticle (NP) in a vacuum simulation box. The box axes are described by a, b, and c.*

1.2. Supported particles on silica

The most stable silica phase at the temperature and pressure commonly employed in catalytic processes, e.g. α-silica, was employed as support.[1] The fully hydrogenated silica(001) surface, the most stable among the low index (001), (111), (110) and (101) surfaces, was employed in this work.[2–4] Silica slabs were generated using the METADISE code.[5] The only difference with the setup employed for isolated particles for DFT calculations is a k-spacing of 0.2 $Å^{-1}$. The α-silica bulk employed for slab construction was optimized via ISIF calculations with the lattice parameters (a=b=4.931 Å, c=5.436 Å, α=β=90°, γ=120°).

Silica slabs of different thicknesses were built containing 3, 6 and 9 SiO_2 units in thickness, as illustrated in **Figure S2**, with a single Pd atom adsorbed. Calculations were performed on (4x4x3), (4x4x6) and (4x4x9) slabs

in order to determine their optimum thickness for prediction of adsorption energies of Pd-based structure on their surface.

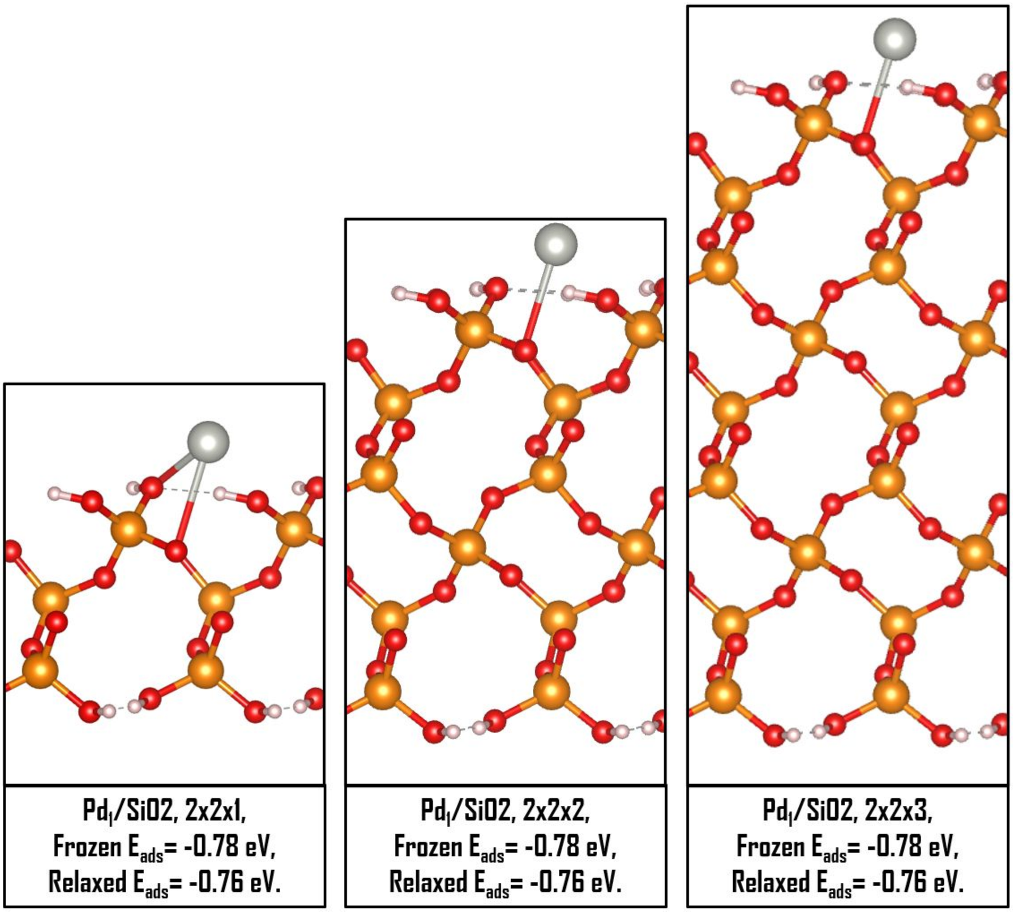

Figure S2: *Illustration and adsorption energies of a single Palladium atom on silica(001) slabs of different thicknesses.*

Initial calculations were performed to determine the extent to which the (4x4x3) slab can be frozen during the relaxation of supported metal structures in order to reduce the computational cost associated with building the dataset employed in this work. Three configurations of Pd_2/silica were explored, with 3 different configurations: Fully frozen silica slab, fully relaxed silica slab and only the surface -OH groups relaxed. The results shown in **Figure S3** reveal that the -OH relaxed configuration provides accurate results compared to the fully relaxed configuration at a much lower computational cost than the entire relaxation. This setup was employed for all supported clusters (size 1 to 8 atoms) explored in this work. Furthermore, other calculations showed that the adsorption energies of supported palladium dimer remained consistent with increasing silica slab thickness. Therefore, the final chosen setup for calculations involving supported metal clusters was formed by a *p(2x2x1)* silica slab.

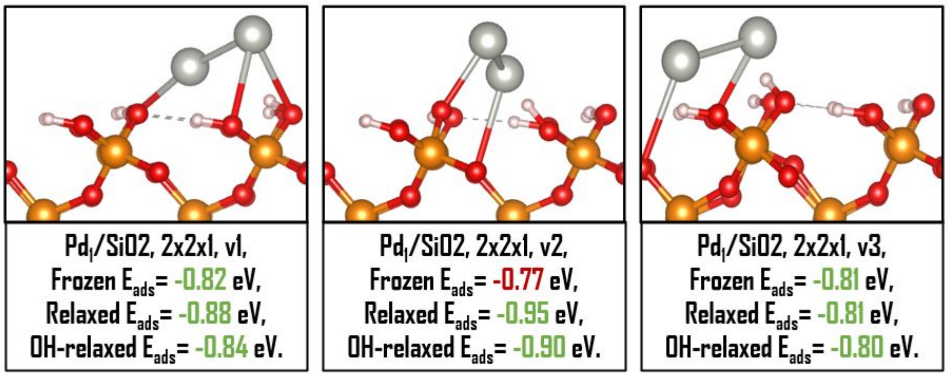

Figure S3: *Illustration and adsorption energies of Pd2 clusters on the (2x2x1)-Silica(001) surface.*

2. Data filters

Two different filters have been applied to the different datasets studied in the present work. The first one consists of a lower threshold to the atomic difference in energy between two successive images found in the dataset. In each optimization trajectory included in the dataset, the energy of image i was compared to that of image $i-1$ according to SI#1.

$$\Delta E = \frac{\mathrm{abs}[(E_i - E_{i-1})]}{n} (SI\#1)$$

Where n is the number of atoms in the system associated with a given optimization path, the obtained value is compared to a selected energy threshold, δE, and accepted in the dataset if $\Delta E > \delta E$. This procedure ensured that the datasets avoided repeating identical and unnecessary images. The impact of such an energy threshold on the dataset is illustrated in the Pd gas-phase dataset shown in **Figure S4**. Applying such a filter significantly reduces the number of points with norm forces between 0.00 and 0.40 eV/Å. Still, it does not influence those belonging to off-equilibrium structures.

The second filter applied to data aimed at preventing outliers from failed electronic convergence iterations whose energies are beyond physically meaningful values. In practice, this filter tested every image selected in the dataset, ensuring that the average atomic energy was within a range bound by two values, E_{min} and E_{max}, which were built differently for each dataset, as shown in **Table S1**.

Dataset	Lower bound, E_{min}	Upper bound, E_{max}

**Pd gas-phase, Pd/SiO2**	$1.00 \times E_{Pd}^{bulk}$	$0.95 \times E_{Pd}^{gas}$
**AuPd gas-phase**	$1.05 \times \frac{E_{Pd}^{bulk} + E_{Au}^{bulk}}{2}$	$0.95 \times \frac{E_{Pd}^{gas} + E_{Au}^{gas}}{2}$

***Table S1**: The energy threshold used to determine potential outliers in the dataset.*

For the Pd gas-phase dataset, the energy of each image in the optimization path was directly compared to the lower and upper bound. For the Pd/SiO_2 dataset, the energy of a naked silica slab was first subtracted from the total energy to remove the contribution from the silica, leaving only the energy of the palladium cluster supported at the surface plus the interaction energy with the surface that was compared to the lower and upper bounds.

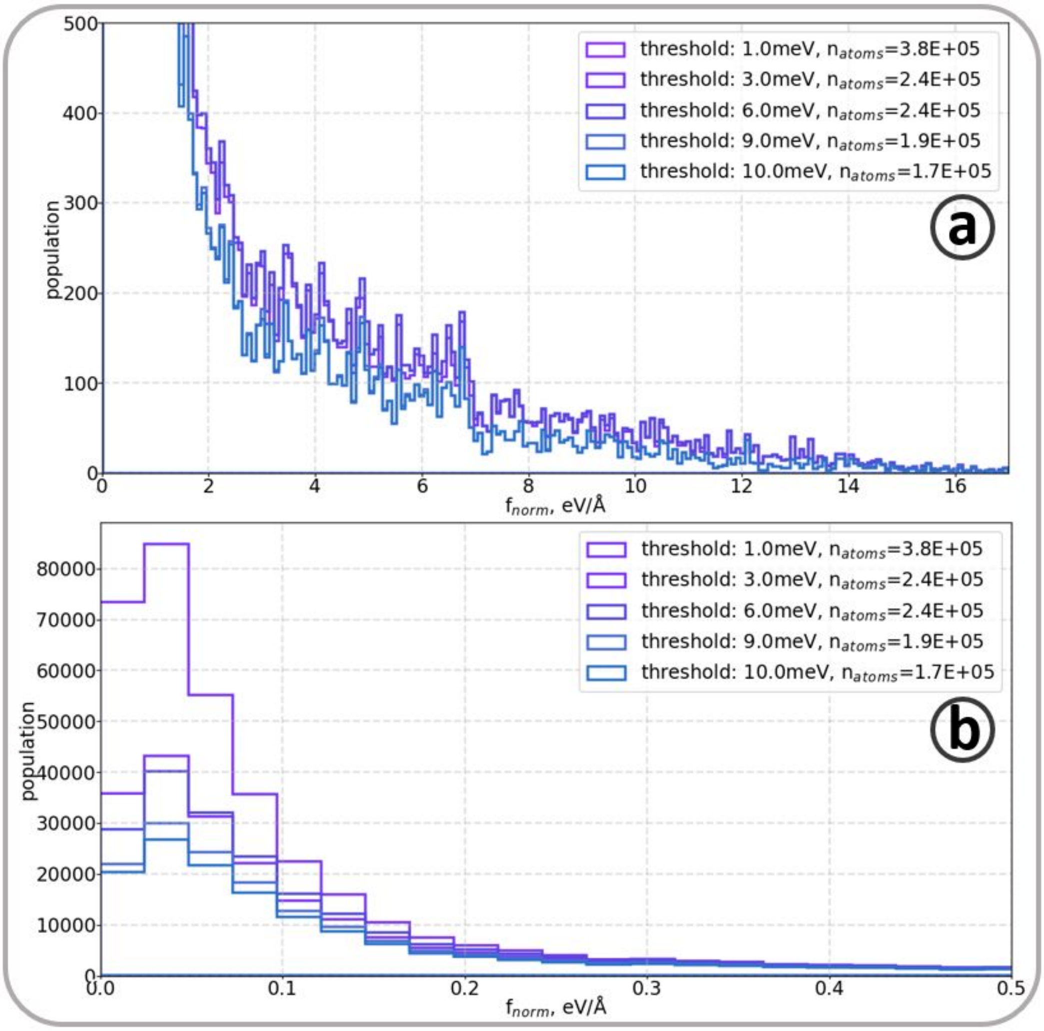

***Figure S4**: (a) Comparison of different filter thresholds (1, 3, 6, and 9 meV) effect on the number of atoms in the Pd-pure dataset for force predicting at small atomic force value (0.00-0.50 eV/Å). (b) Zoom in on the near-zero forces region, i.e. the range [0.0, 0.5] eV/ Å.*

3. Pre-processing: Principal component analysis and auto-encoding

Two pre-processing approaches were tested to predict forces norms: Principal component analysis and auto-encoders. Principal Component Analysis (PCA) is a popular pre-processing method. PCA starts with computing the covariance matrix of the data $\boldsymbol{X}$. This squared matrix stores the covariance between each pair of elements of the matrix $\boldsymbol{X}$. The (i, j) component of the covariance matrix will contain the covariance between dimension i and dimension j. The covariance of two elements in a column vector $\boldsymbol{x} = [x_1, x_2,...,x_N]^T$ is computed through Eq. (SI#2).

$$cov[x_i,x_j] = (x_i - \langle x_i \rangle) \cdot \left(x_j - \langle x_j \rangle\right) (SI\#2)$$

Where $\langle x_i \rangle$ and $\langle x_j \rangle$ are the average expected values of parameters x_i and x_j. A positive covariance means that the two elements are related. A negative covariance means that two elements are negatively related and would tend to get more and more 'different' from each other. The diagonal element of the covariance matrix contains information on the variance of the element x_i, informing how far the element d_i deviates from its average value over the entire dataset, calculated using Eq. (SI#3).

$$cov[x_i,x_i] = (x_i - \langle x_i \rangle) \cdot (x_i - \langle x_i \rangle) = Var(d_i) (SI\#3)$$

Once the covariance matrix is computed, it can be demonstrated that this matrix can be decomposed (*Singular Value Decomposition*) via Eq. (SI#4).

$$\boldsymbol{\Gamma} = \boldsymbol{U} \cdot \boldsymbol{S} \cdot \boldsymbol{V} (SI\#4)$$

Where $\boldsymbol{\Gamma}$ is the covariance matrix, and $\boldsymbol{U}$ is a matrix whose columns form a set of linearly independent vectors. The dot product of this matrix $\boldsymbol{U}$ with the initial matrix, $\boldsymbol{X}$, containing the dataset provides the dimension-reduced PCA *dataset*.

$$\boldsymbol{X_{PCA}} = \boldsymbol{X} \cdot (SI\#5)$$

Auto-encoders (AE) generate a n-layer coding of an input vector. The structure of an AE can be divided into three distinct parts: The encoder, the decoder, and the latent space. The encoder generates compressed data with a dimension usually lower than the input vector. The decoder reproduces the initial input vector from the compressed data. The encoded input is generated in the latent space. AE usually have a mirror structure, as illustrated in **Figure S5**.

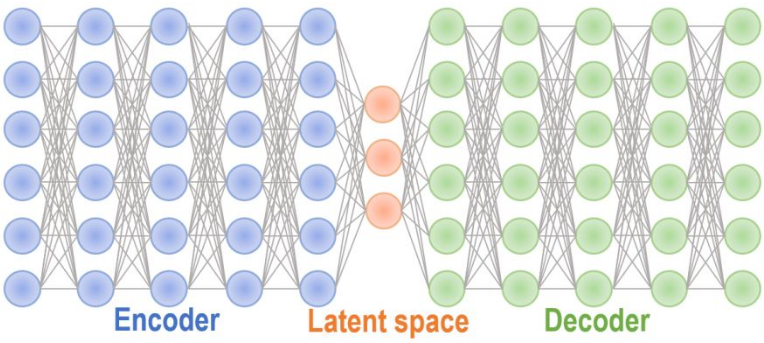

Figure S5: *Illustration of the architecture of an Auto-Encoder.*

Considering an AE where the encoder contains two hidden later, the dimension-reduced vector obtained in the latent space is given by Eq. (SI#6).

$$\boldsymbol{h_z} = f_e(g_e(\boldsymbol{x}))(SI\#6)$$

Where g_e represents the first hidden layer, f_e the second hidden layer, $\boldsymbol{x}$ is the input (high-dimensional) vector, and $\boldsymbol{h}$ is the dimension-reduced vector obtained in the latent space. The decoder is a feed-forward NN taking the dimension-reduced vector as input and trying to reproduce the original input vector according to Eq. (SI#7).

$$\overline{\boldsymbol{x}} = g_d(f_d(\boldsymbol{h}))(SI\#7)$$

Where now, f_d represents the first hidden layer and g_d is the second hidden layer. $\overline{\boldsymbol{x}}$ is the vector produced by the decoded. The closer the reproduced vector $\overline{\boldsymbol{x}}$ is from the original input $\boldsymbol{x}$ the better the AE performs. An example of the loss function employed in these encoders is the L2 loss given in Eq. (SI#8).

$$\mathcal{L}(\boldsymbol{x},\overline{\boldsymbol{x}}) = |\overline{\boldsymbol{x}} - \boldsymbol{x}|^2 = \sum_j (\overline{x}_j - x_j)^2(SI\#8)$$

The advantage of using an auto-encoder over other methods, such as Principal Component Analysis (PCA) for dimension reduction, is that PCA is a linear dimension reduction method. It relies on capturing the variance of the dataset in hyperplanes by building linear components that correlate with the original data. In real-life problems, most correlations don't follow linear relationships, which auto-encoders can capture.

The performance of an AE to encode and decode an input vector can be evaluated through the cosine distance between the input, $\boldsymbol{x}$, and the output, $\overline{\boldsymbol{x}}$ according to Eq. (SI#9).

$$D_{cos}(\boldsymbol{x},\overline{\boldsymbol{x}}) = 1 - S_c(\boldsymbol{x},\overline{\boldsymbol{x}}) = 1 - \frac{\boldsymbol{x} \cdot \overline{\boldsymbol{x}}}{||\boldsymbol{x}|| \cdot ||\overline{\boldsymbol{x}}||}(SI\#9)$$

The cosine distance has values in the range [0, 1]. 1 means the two vectors are orthogonal, i.e., are not similar in any way. A cosine distance of 0 means the two vectors are identical.

4. Performance: PCA vs. AE

For quantitative assessment of the performance of PCA vs AE, a specific dataset was designed, containing only supported particles on silica, using an energy threshold of 0.001 meV/atom between two images allowed in the dataset. These settings led to a dataset containing around 200,000 atoms. A 60D fingerprint was collected to describe these systems, covering only the forces norm: Feature engineering proved unnecessary for energy or forces-direction predictions. This smaller fingerprint is justified by the desire to produce a quantitative assessment between the two methods rather than a qualitative evaluation. The fingerprint extracted from Behler's symmetry functions is described in **Table S2,** and the fingerprint extracted from perturbed symmetry functions is described in **Table** .

	Weight	R_s (Å)	η (Å²)	λ (∅)	ξ (∅)
**G^2**	[2, 3, 5, 7]	[3.0, 3.5, 4.0, 4.5, 5.0, 5.5, 6.0]	[1.0, 3.0, 6.0]	∅	∅
**G^3**	[2, 3, 5, 7]	∅	∅	[-1, +1]	[2.0, 8.0, 16.0]

Table S2: *Table reporting the parameters employed to build the pure palladium gas-phase energy fingerprint.*

	Weight	η (Å²)	Cheby. Deg.	λ	ξ	Pseudo. Cheby. Deg.
$\tilde{G}^2$	[2, 3, 5, 7]	[1.0, 3.0, 6.0]	[2, 4, 6]	∅	∅	∅
$\tilde{G}^3$	[2, 3, 5, 7]	∅	∅	[-1, +1]	[1.0, 8.0, 16.0]	[3, 4, 5]

Table S3: *Table reporting the parameters employed to build the pure palladium gas-phase forces-norm fingerprint.*

Training an AE to reduce the dimension of the 60D fingerprint to any dimension in the range [30, 55] is straightforward for a simple AE within 200 epochs. As illustrated in **Figure S6**, the test-set RMSE reaches the value 0.009, and the mean cosine distance reaches 0.000, meaning the AE performs the encoding-decoding operation with neglectable mistakes while reducing the dimension of the fingerprint from 60D to any value in the range [30, 55]. **Table S44** reports the structure employed by the AE in this section. The structure of the feed-forward NN learning the forces norm from dimension-reduced fingerprints is described in **Table S5**. To

keep the results obtained easily comparable, that structure was identical to the overall AE training performed in this work, independent of the fingerprint size.

NN structure	Optimizer	Scheduler	Other parameters
Encoder (200, 60, 60); lattent space: 30 to 55; Decoder (60, 60, 200). Lattent space transformation: Sigmoid. Activation function: Leaky-ReLU (slope: 0.02)	**NAdam**; learning rate: 0.7×10^{-3};. **200 epochs**. L2-regularization: 0.2×10^{-4}. **Foreach** enabled.	Multi-step scheduler at 70% and 90% epochs. γ=0.2	Weights: Xavier normal, bias: $\mathcal{N}(\mu = 0.0, \sigma = 1.0)$.

Table S4: *Structure of the Encoder trained for supported particles' input encoding.*

NN structure	Optimizer	Scheduler	Other parameters
Feed-forward NN (80, 40, 40, 1); Activation function: Leaky-ReLU (slope: 0.05)	**NAdam**; learning rate: 1×10^{-3};. **1200 epochs**. L2-regularization: 0.1×10^{-3}. **Foreach** enabled.	Triangular learning-rate. Min: 1×10^{-4}. Max: 1×10^{-3}. 400 epoch/step.	Weights: Xavier normal, bias: $\mathcal{N}(\mu = 0.0, \sigma = 1.0)$.

Table S5: *Structure of the NN trained for supported particles' forces norm predictions in this chapter.*

The results obtained after the reduction of the initial 60D fingerprint are shown in **Table** and illustrated in **Figure S6** (b). The results obtained by reducing the input's dimension through principal component analysis (PCA) are always better by 0.02 to 0.03 eV/Å on average. This difference cannot be imputed to the encoder (AE) failing: The cosine distance obtained by training AE is always inferior to 0.005 for any dimension in the range [20, 55]. Although, in theory, the AE finds complex relationships between the input elements, it appears that the PCA produces better results in the present case. Therefore, we employed this feature engineering method for the forces norm prediction of the total dataset, including supported silica and gas-phase clusters.

Reduced fingerprint dimension	MAE, post-training on PCA, eV/Å	MAE, post-training on AE, eV/Å
**20**	0.089	0.104
**25**	0.081	0.114
**30**	0.072	0.104
**35**	0.072	0.116
**40**	0.070	0.121
**45**	0.073	0.109
**50**	0.078	0.101
**55**	0.074	0.109

Table S6: *Compared performance of a neural network training to learn atomic forces norm with the fingerprint of the same dimension, reduced using PCA or AE.*

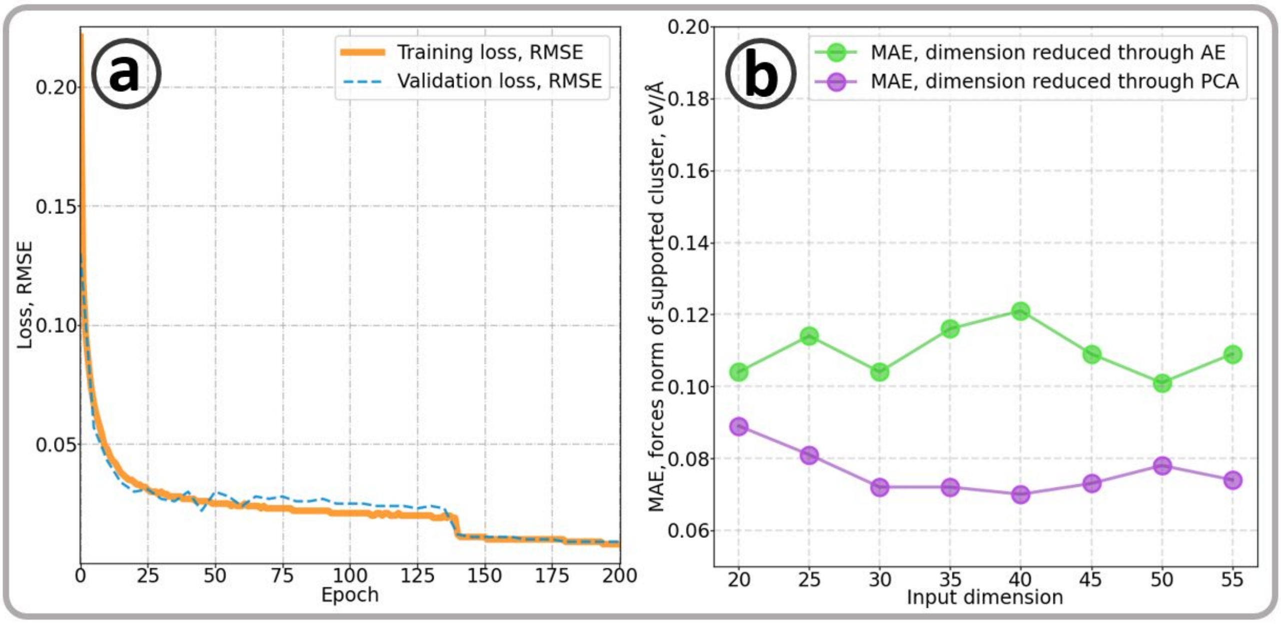

Figure S6: *(a) Learning curve of the auto-encoder (AE) reducing the dimension of the forces norm fingerprint from 60D to 30D. (b) Performance of the neural network predicting the atomic forces norm with different fingerprint dimensions reduced through auto-encoding (AE, Green) or principal component analysis (PCA, Purple).*

5. Reference

1. Keskar, N. R. & Chelikowsky, J. R. *Structural Properties of Nine Silica Polymorphs*. vol. 46 (1991).

2. Murashov, V. V. Reconstruction of pristine and hydrolyzed quartz surfaces. *Journal of Physical Chemistry B* **109**, 4144–4151 (2005).

3. Goumans, T. P. M., Wander, A., Brown, W. A. & Catlow, C. R. A. Structure and stability of the (001) α-quartz surface. *Physical Chemistry Chemical Physics* **9**, 2146–2152 (2007).

4. Schlexer, P. & Pacchioni, G. Adsorption and Dimerization of Late Transition Metal Atoms on the Regular and Defective Quartz (001) Surface. *Top Catal* **60**, 459–470 (2017).

5. Watson, G. W., Kelsey, E Toby, De Leeuw, N. H., Harris, D. J. & Parker1, S. C. *Atomistic Simulation of Dislocations, Surfaces and Interfaces in MgO*.
